# Approaching infinite selectivity in membrane-based aqueous lithium extraction via solid-state ion transport

**DOI:** 10.1126/sciadv.adq9823

**Published:** 2025-02-28

**Authors:** Sohum K. Patel, Arpita Iddya, Weiyi Pan, Jianhao Qian, Menachem Elimelech

**Affiliations:** Department of Chemical and Environmental Engineering, Yale University, New Haven, CT 06520-8286, USA.

## Abstract

As the gap between lithium supply and demand continues to widen, the need to develop ion-selective technologies, which can efficiently extract lithium from unconventional water sources, grows increasingly crucial. In this study, we investigated the fundamentals of applying a solid-state electrolyte (SSE), typically used in battery technologies, as a membrane material for aqueous lithium extraction. We find that the anhydrous hopping of lithium ions through the ordered and confined SSE lattice is highly distinct from ion migration through the hydrated free volumes of conventional nanoporous membranes, thus culminating in unique membrane transport properties. Notably, we reveal that the SSE provides unparalleled performance with respect to ion-ion selectivity, consistently demonstrating lithium ion selectivity values that are immeasurable by even the part-per-billion detection limit of mass spectrometry. Such exceptional selectivity is shown to be the result of the characteristic size and charge exclusion mechanisms of solid-state ion transport, which may be leveraged in the design of next-generation membranes for resource recovery.

## Teaser

Solid-state transport enables near-perfect lithium ion selectivity, inspiring next-generation ion-selective membrane design.

## INTRODUCTION

As global decarbonization efforts continue to gain momentum, it is projected that more than half of worldwide vehicle sales will be electric by the year 2035 (*1*). While such widespread electrification of the transportation sector is advantageous in reducing overall greenhouse gas emissions, the rapid transition to electric vehicles (EVs) is expected to place severe strain on the supply chain of critical elements used in EV batteries (*2*). Because of their low cost and unparalleled energy density (*3*), lithium-ion batteries are expected to remain the dominant battery chemistry for EVs. While the need for some critical elements in lithium-ion batteries (e.g., cobalt and nickel) may potentially be eliminated through the development of alternate cathode materials, lithium remains an essential component which is required in substantial quantities (i.e., ~8 kg in a single EV battery pack) (*1*, *2*). Accordingly, the demand for lithium is anticipated to grow exponentially over the next decade, reaching values beyond what can be attained from conventional sources (i.e., mining of lithium ores or extraction from lithium rich brines) by the year 2030 (*4*). Hence, effectively addressing this lithium supply gap will require extraction of lithium from unconventional aqueous sources (e.g., groundwaters, oil and gas produced water, industrial wastewaters, and geothermal brines) (*5*–*8*).

The conventional strategy to extract lithium from aqueous sources relies on pre-concentration via solar evaporation, followed by a series of chemical-based purification and precipitation steps (*9*, *10*). Hence, lithium extraction is currently geographically limited to arid regions with ample land, requires long processing times, has adverse environmental impacts (i.e., chemical and freshwater consumption), and suffers from low lithium recovery rates (*9*, *11*). The development of direct lithium extraction (DLE) technologies, which are capable of circumventing time- and land-intensive pre-concentration steps and which can attain high purity lithium without the need for chemical based posttreatment steps, has therefore been extensively studied in recent years. While ion-exchange resins and adsorbents have been highly investigated for lithium extraction, such methods still require partial pre-concentration of lithium due to their limited lithium selectivity (over competing cations) and necessitate large volumes of freshwater or chemicals to regenerate. In contrast, electrochemical lithium intercalation, in which lithium ions are capacitively stored in layered or lattice structures (most commonly battery electrodes), has demonstrated impressive lithium selectivity (over both magnesium and sodium) (*12*–*14*). Nonetheless, intercalation-based approaches now suffer from severely limited electrode life span and require periodic regeneration, inherently requiring semi-batch operation (*15*).

Highly selective membranes, which facilitate the preferential transport of lithium over competing species, have the potential to overcome the limitations of ion-exchange and intercalation-based approaches by providing continuous and sustainable lithium recovery. Considerable research efforts towards the development of lithium-selective membranes have culminated in materials that are capable of strongly distinguishing between lithium and magnesium, primarily by exploiting differences in ion valency, hydrated size, and hydration energy (*7*, *16*). However, the effective separation of monovalent ions, such as lithium and sodium, has proven markedly more challenging, with little to no selectivity being realized in most of the synthetic membranes and nanochannels (*17*–*24*). As typical brines contain substantially higher concentrations of sodium compared to lithium, the practical effectiveness of DLE using current state-of-the art membranes is limited. Thus, the continued investigation of membrane materials that provide high lithium selectivity against both commonly competing divalent and monovalent ions remains critical.

Motivated by safety concerns stemming from the flammability of commonly used liquid electrolytes, the batteries field has increasingly focused on the development of solid-state electrolytes (SSEs), rigid three-dimensional cation-anion frameworks that allow for the migration of a mobile cation (e.g., lithium) (*25*). In these solid materials, the transport of a mobile cation is facilitated through the migration of defects (i.e., vacancy or interstitial sites) in the crystalline structure (*26*, *27*). Although SSEs have become a highly investigated research area in the battery community, culminating in the development of highly conductive materials, limited attention has been given toward their potential application as a unique class of membrane materials for aqueous ion separations. While a few proof-of-concept studies have demonstrated that SSE materials could be used for selective extraction of lithium ions from seawater (*28*–*30*), fundamental evaluation and understanding of transport in SSEs applied to aqueous systems remains unexplored.

In this study, we systematically assess the application of a lithium ion–conducting SSE as a membrane for aqueous lithium extraction. We begin by investigating the fundamentals of ion and water transport in the SSE while providing direct comparison to a conventional cation-exchange membrane to highlight the unique mechanisms of solid-state diffusion. Upon gaining an understanding of transport in the SSE with a single-salt system, we evaluate the ion-ion selectivity of the SSE against commonly competing cations in lithium brines. Our results reveal immeasurably low fluxes for competing salt ions over all investigated conditions, indicating virtually perfect lithium selectivity. Nonetheless, we also uncover an important relationship, whereby the presence of competing cations, despite being impermeable, still adversely affects the flux of lithium ions. By applying experimental characterization techniques in conjunction with molecular dynamics simulations, we provide mechanistic insights into the atypical ion-ion selectivity phenomena observed with the SSE. We conclude by considering the practical implications of the observed SSE performance and by highlighting critical research directions for the continued development of SSE materials as ion-selective membranes in aqueous separations.

## RESULTS

### Fundamentals of lithium and water transport in SSEs

**Fig. 1.**
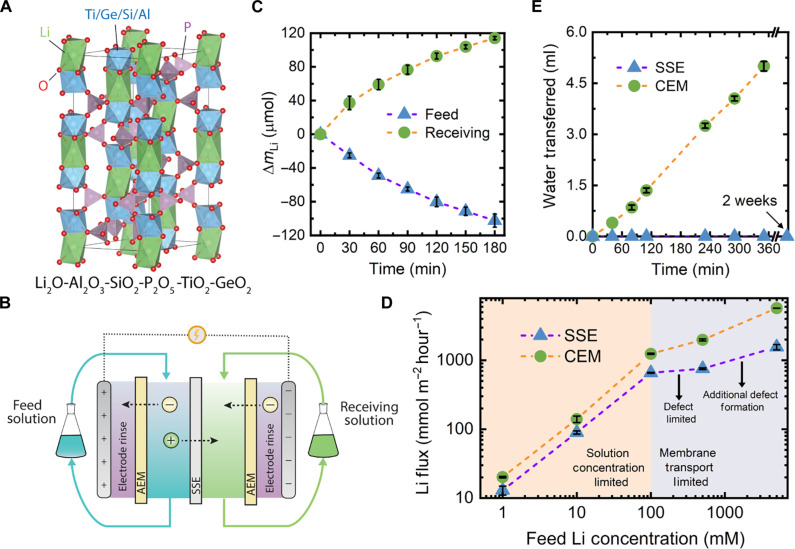
Investigation of ion and water transport in the SSE. (**A**) The crystalline lattice of the NASICON-like SSE material used throughout this study, as visualized in VESTA (*70*). The rhombohedral unit cell consists of phosphate tetrahedra (purple) that share corners with either titanium, germanium, aluminum, or silicon octahedra (blue). Lithium ions form octahedra in the lattice sites (green), although they may also occupy less energetically favorable tetrahedra interstitial sites during ion migration. (**B**) The process schematic of the batch electrodialysis (ED) setup used to evaluate ion transport under an applied electric field. The feed and receiving solutions flow across the SSE [or cation exchange membrane (CEM)] via the inner flow channels, while the external flow channels serve as electrode rinse compartments. The feed and receiving solutions remained hydraulically disconnected, whereas a single-electrode rinse solution was recirculated through both electrode rinse chambers. (**C**) Lithium ion mass balance in batch ED experiments using the SSE. The change in the amount of lithium (Δ*m*_Li_) in the feed and receiving solution was monitored while a constant 4 V potential was applied for 3 hours. The feed and receiving solutions initially consisted of 10 mM LiCl and 10 mM KCl, respectively. (**D**) The effect of feed solution lithium concentration on the lithium flux for the SSE and CEM. In each of the experiments, the receiving solution was 10 mM KCl, while the concentration of LiCl in the feed solution was varied. A constant potential of 4 V was applied throughout each experiment. On the basis of the flux response from both types of membranes, the feed concentration range is broken into two transport regimes, where the lithium flux is either solution concentration limited or membrane transport limited. (**E**) Assessment of water transport across the SSE and CEM in a diffusion cell. The feed chamber consisted of deionized water and the receiving chamber consisted of 500 mM sucrose.

Although SSEs have been extensively studied in solid-state batteries, such materials have yet to be thoroughly assessed as membrane materials for aqueous separations. Hence, we begin our study by systematically exploring the transport of water and lithium ions between two aqueous solutions separated by an SSE. Throughout this study, we used a commercially available lithium ion–conducting NASICON-type SSE with the structure shown in Fig. 1A. Specifically, the rhombohedral crystal lattice is a doped variant of LiTi_2_(PO_4_)_3_, in which a portion of the titanium atoms have been substituted for germanium, aluminum, or silicon to enhance the ionic conductivity (*25*, *31*). Nonetheless, the typical NASICON-type crystal structure is maintained, whereby phosphate tetrahedra share corners with metal (titanium, germanium, aluminum, or silicon) octahedra and mobile lithium ions occupy either octahedral lattice sites or tetrahedral interstitial sites. Notably, this particular NASICON-type SSE was selected because of its high lithium ion conductivity and exceptional water stability, in contrast to most other SSE material classes that readily decompose in the presence of water (*25*, *32*).

It is well established in the literature that transport across the crystalline framework of an SSE occurs via hopping of the mobile cations across point defects, either in the form of lattice site vacancies or through the insertion of ions into interstitial sites (*25*). In battery systems, the required energy to induce such defects is provided via an electric field driving force. However, membrane processes may also use various other driving forces for mass transport, such as a pressure or concentration gradient. Thus, we began our evaluation of the SSE by investigating the permeability for lithium ions via pure diffusion. However, throughout such diffusion experiments (details in the Supplementary Materials), no lithium flux was detected across the SSE, even in the presence of a 500 mM lithium ion transmembrane concentration difference (fig. S1). It is important to note that, in the absence of an electric field, the condition of charge neutrality in each solution requires that lithium ions diffuse across the SSE alongside an anion (i.e., chloride ions). Hence, we surmise that the lack of concentration gradient–driven lithium transport arises from the exclusion of anions by the SSE. Specifically, while the SSE structure accommodates the migration of lithium ions, the insertion of chloride ions (or other anions) into the crystalline structure is expected to be highly unfavorable due to their larger ionic size and opposite valency. Accordingly, we determine that SSE materials are not likely to be practical in concentration gradient– or pressure-driven applications, where the flux of cations and anions is coupled. Additionally, inorganic SSE materials are unsuitable for pressure-driven applications, as they are generally mechanically inflexible and brittle (*25*). Thus, for the remainder of this study, we assess ion transport across the SSE solely under an applied electric field.

Upon incorporating the SSE into an electrodialysis (ED) cell (Fig. 1B and fig. S2) and applying a constant cell potential of 4 V, the concentration of lithium ions in the receiving compartment was found to continuously increase over time, while the concentration of other cations in the receiving solution (i.e., K^+^, Mg^2+^, and H^+^) remained relatively invariable (fig. S3). Thus, it was determined that lithium is the primary cationic charge carrier. However, with the SSE framework inherently consisting of lithium ions, it is necessary to ensure that the observed lithium in the receiving solution is not a result of leaching from the material.

To identify the source of the accumulating lithium ions in the receiving solution, we performed a mass balance across the feed and receiving solutions. As shown in Fig. 1C, over the 3-hour duration of applying a potential, the lithium concentration in the feed solution decreases, while the lithium concentration in the receiving solution increases. This mirroring of concentration change across the feed and receiving solutions assures that lithium ions are not being depleted from the SSE material but are rather migrating through it (i.e., from the feed solution to the receiving solution). While the magnitudes of the concentration change in the feed and receiving solutions align closely, we find that the amount of lithium in the receiving solution is consistently ~12 μmol greater than the feed solution. Notably, this difference is found to emerge within the first 20 min of applying a potential, after which the change in concentration between the solutions is found to be nearly equal and opposite. Thus, we attribute the initial divergence to start-up phenomena, such as the release of surface adsorbed lithium ions on the SSE.

Next, we aimed to gain further insight into ion transport through the SSE by evaluating how the lithium flux is affected by the external solution concentration of lithium ions. Hence, while the composition of the feed solution remained solely lithium chloride (LiCl) throughout, the concentration of LiCl was varied over orders of magnitude (i.e., 1 mM to 5000 mM LiCl). To contextualize the magnitude of the lithium flux in the SSE, we also performed the same set of experiments with a conventional cation exchange membrane (CEM). As shown in Fig. 1D, the lithium flux for both the SSE (blue points and purple line) and CEM (green points and orange line) show a clear dependence on the external solution concentration, generally increasing with the feed solution concentration. Notably, over the entire concentration range assessed, the lithium flux for the SSE remained below that of the CEM, albeit to varying extents depending on the solution concentration. As the membrane was the only variable that was changed across these experiments, we conclude that the SSE poses a higher transport resistance compared to the CEM (further discussion provided in the following section).

For both the SSE and CEM, the dependence of the lithium flux on external solution concentration can be broken into two distinct regimes. Specifically, as the concentration is increased from 1 mM LiCl up to 100 mM LiCl, the lithium flux for both membrane types follows nearly the same power relation, indicating that similar phenomena are likely limiting the attainable flux. At lithium chloride concentrations >100 mM, however, this relationship no longer holds, and the lithium fluxes for the CEM and SSE begin to diverge more substantially. Hence, the similar power law relation observed for both the SSE and the CEM in the lower concentration regime is expected to be related to the effects of the low electrolyte concentration. Specifically, at such low salt concentrations, the solution resistance and diffusion boundary layer resistance at the membrane-solution interface are likely to be dominant over the membrane resistance (*33*, *34*). Therefore, in this regime, the lithium flux is highly sensitive to the feed solution concentration, whereby increasing the solution concentration by an order of magnitude also correlates to nearly an order of magnitude enhancement of lithium flux.

In contrast, at concentrations >100 mM LiCl, the solution resistance and diffusion boundary layer resistance are effectively minimized, leading to decreased sensitivity of the lithium flux to solution concentration. Hence, in this regime, the lithium flux is expected to be membrane transport limited, making differences between the transport mechanisms of the SSE and CEM more apparent. Specifically, as the solution concentration is progressively increased within this regime, the lithium flux through the CEM consistently increases, while, in the case of the SSE, the lithium flux demonstrates two distinct growth rates (i.e., an initial plateau followed by continued increase in flux).

The differing observed solution concentration dependence of the CEM and SSE may be understood by considering the properties which dictate the ionic conductivity of each type of membrane. With ion-exchange membranes, increasing the external solution concentration leads to gradual screening of the membrane’s fixed charge and thus weakened Donnan exclusion. Consequently, more co-ions are introduced into the membrane matrix, which, due to charge neutrality, must be accompanied by an increased number of counterions (i.e., lithium ions). This increased total ion concentration in the membrane effectively leads to higher ionic conductivity at the expense of greater salt leakage (i.e., decreased permselectivity) (*35*). Notably, these effects are consistently magnified as the solution concentration is increased, in agreement with the observed rate of growth in the lithium flux.

As with an ion-exchange membrane, the conductivity of an SSE is directly related to the number of mobile charge carriers within the material. However, the number of lithium ions that may be accommodated in an SSE remains primarily fixed by the crystalline structure and the condition of charge neutrality. Thus, in a crystalline SSE, the number of mobile charge carriers, rather than being the number of lithium ions, is more accurately interpreted as the number of point defects (i.e., cationic vacancies and occupied interstitial sites). With the formation of these point defects being an activated process (*25*), increasing the chemical potential energy of the system (i.e., by increasing the feed solution concentration) may induce the formation of a larger number of defects, thus opening additional ion migration pathways and increasing the SSE conductivity. However, a notable increase in the growth rate of the flux is only observed when an extreme LiCl concentration of 5000 mM is used, implying that, within practical solution concentration ranges, the number of available defects (and, hence, the conductivity) in the SSE remains relatively fixed, thus limiting the attainable lithium flux. It should also be noted that, while the SSE conductivity may be increased through inducing a larger amount of point defects, excessive defect formation could lead to destabilization of the lattice structure and eventual degradation of the SSE.

While the SSE is confirmed to serve as a conductor for lithium ions, aqueous separations also inherently involve water molecules, which may interact with or traverse the SSE. To evaluate the interaction of the SSE with water molecules, water uptake experiments were conducted, in which the mass of several SSE fragments was compared before and after long-term exposure to water. Overall, no change in the mass was observed, indicating that the material does not readily absorb water or decompose in the presence of water (fig. S4). Furthermore, we evaluated the permeability of water through the SSE material by completing a series of osmosis experiments, in which the SSE separated a concentrated (500 mM) sucrose solution from deionized water (fig. S5). Although the large osmotic pressure difference (~12 bar) between the two solutions provides a substantial driving force for water to traverse the membrane, no change in the volume was observed over the course of 2 weeks (Fig. 1E). In contrast, when the SSE was replaced with a CEM, a steady water flux was observed within just 1 hour.

The impermeability of the SSE to water is likely due to water molecules not being able to penetrate the rigid and tightly packed NASICON crystalline lattice (Fig. 1A). Specifically, analysis of the crystalline lattice of LiTi_2_(PO_4_)_3_, a close analog of the SSE used in this study (i.e., without doping), reveals that the distance between many oxygen atoms is less than the diameter of a water molecule (i.e., 2.7 Å), thus preventing access into the structure. We further confirmed the inability of water molecules to enter the crystalline lattice by executing PoreBlazer (*36*) simulations on the LiTi_2_(PO_4_)_3_ unit cell using a 2.7-Å-sized probe. The calculations determined that, although the interstitial space in LiTi_2_(PO_4_)_3_ has a total volume of 286.2 Å^3^, none of the free volume is accessible to the probe, thus supporting our experimental findings that water molecules are unable to enter the SSE structure. This conclusion implies that ion transport in the SSE occurs solely under anhydrous conditions, in which ions are fully stripped of their hydration shell.

### Unique transport energy barriers posed by solid-state ion migration

While in the prior section, the flux of lithium ions across the SSE was determined over various conditions, the fundamental intrinsic membrane properties, which facilitate more meaningful and direct comparison between materials, were not assessed. Here, we begin by quantifying the ionic conductivity of the SSE and CEM to gauge the relative ease with which a lithium ion may traverse each type of material (fig. S7). To ensure that the measured conductivity is primarily reflective of transport across the membrane and does not include considerable contributions from external resistances (i.e., solution resistance and diffusion boundary layers), a high-salt concentration (500 mM LiCl) solution was used, and vigorous mixing of the solution in each compartment was provided. Nonetheless, we found the conductivity of the SSE to be 0.05 mS cm^−1^, approximately one-half of the reported conductivity by the manufacturer. Such discrepancy may be attributed to our measurement of the SSE conductivity in an aqueous system, as opposed to the typical method of testing SSE conductivity in a solid-state battery cell. Specifically, in an aqueous system, it is likely that the ion dehydration required for lithium to partition from solution into the SSE imposes a larger overall transport resistance compared to the deintercalation of (already dehydrated) lithium ions in battery electrodes, thus leading to a lower practically measured conductivity.

**Fig. 2.**
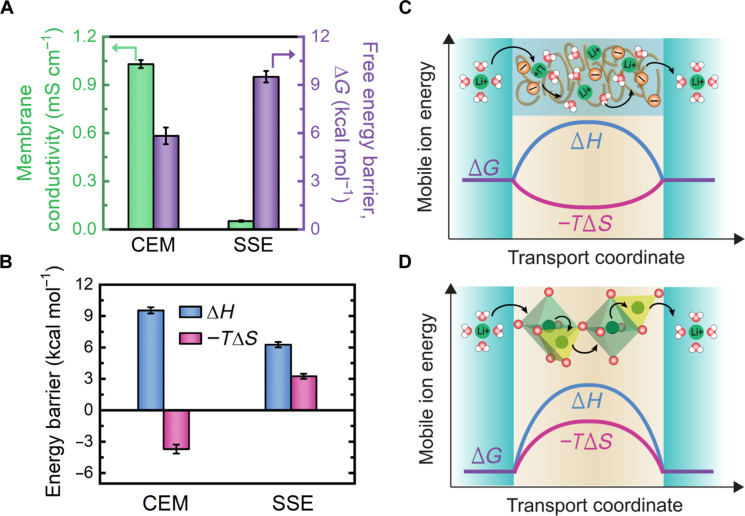
Fundamental transport differences between an SSE and CEM. (**A**) Comparison of membrane conductivity (green bars) and free energy barrier (purple bars) for the CEM and SSE. The conductivity values shown are obtained by measuring the potential difference across the membrane at various applied current densities at room temperature. Free energy barriers for lithium transport are determined through the measurement of membrane conductivity at various temperatures. (**B**) The enthalpic (blue bars) and entropic (pink bars) contributions to the free energy barrier for the CEM and SSE. Schematic illustration highlighting the trends of the free energy coordinate for the (**C**) CEM and (**D**) SSE as lithium ions are transported from the feed side, through the membrane, and into the receiving solution. The distinct mechanisms of transport are illustrated for each membrane type. In the CEM, the ions interact with the negative fixed-charge groups in the membrane while traversing tortuous hydrated free volume elements between polymer chains. In the SSE, the lithium ions undergo dehydration as they partition into the SSE while simultaneously being stabilized by the oxygen atoms in the lattice. The dehydrated lithium ions undergo single-file hopping through octahedral and tetrahedral sites as they migrate across the crystalline structure. The relative entropic (pink) and enthalpic (blue) barriers for transport are shown for the mechanisms of transport in the CEM and SSE.

In comparison to the SSE, the CEM was found to have a substantially higher ionic conductivity (Fig. 2A). Specifically, we measured the lithium conductivity of the CEM to be nearly 20 times greater than that of the SSE, emphasizing the distinct modes of transport in each type of membrane. The higher conductivity of the CEM may be rationalized by considering that ions are considerably more mobile in large (nanometer-scale) water-filled channels as compared to in highly confined crystalline solids (*37*, *38*). Whereas the tight and rigid packing of atoms in the SSE structure does not allow for the penetration of water molecules and thus only facilitates ion transport through classical solid-state diffusion mechanisms, ion-exchange membranes are composed of flexible polymer chains that swell under aqueous conditions, effectively forming interconnected water channels through which ions migrate (*38*–*40*). Hence, unlike the SSE, ion transport in the CEM may be interpreted as tortuous (i.e., between randomly oriented polymer chains) and hindered transport (i.e., due to interactions with fixed-charge groups) through a liquid phase (*39*).

Although the conductivity is a useful intrinsic property for comparing membrane performance, it is insufficient for describing the molecular level interactions between the mobile ion and the membrane, which ultimately dictate transport. Transition state theory, in contrast, provides greater fundamental insight by assuming that the transport of an ion through a membrane can be perceived as hopping among equilibrium states at various energy levels. Each of these attempted hops is thus associated with an energy barrier which may originate from various molecular-level phenomena such as ion dehydration, steric hindrance, or interaction of ions with moieties in the membrane structure (*41*–*43*). Hence, to elucidate the fundamental differences between transport in the SSE and CEM, the energy barrier of lithium ion permeation for each membrane was determined (Fig. 2A and fig. S8).

The overall energy barrier for lithium transport through the SSE was found to be higher than that of the CEM by 3.7 kcal mol^−1^, in good agreement with the measured (room temperature) conductivity (Fig. 2A). Nonetheless, to draw further insight into the mechanistic differences in transport through the SSE and CEM, the respective enthalpic and entropic contributions must be compared. As the enthalpic barrier reflects the specific interactions between the ion and the membrane during ion partitioning and transmembrane transport, it is often associated with phenomena such as ion dehydration, electrostatic interactions, and ion-ligand binding within the membrane (*41*). Hence, it could intuitively be expected that the SSE should have a larger enthalpic penalty compared to the CEM because partitioning of lithium ions into the SSE requires complete dehydration. Nonetheless, we find the activation enthalpy for the SSE to be 3.3 kcal mol^−1^ smaller than that of the CEM (Fig. 2B). To understand such a result, it is critical to note that shedding of the hydration shell is accompanied by simultaneous interactions with moieties of the membrane, which can effectively stabilize the ion and offset the energetic penalty of ion dehydration (*44*).

In aqueous solution, lithium ions are stabilized via ion-dipole interactions with the electronegative oxygen atoms of water molecules, typically forming a tetrahedral configuration with four water molecules (*45*–*47*). Notably, in the NASICON-like SSE used in this study, lithium ions are similarly stabilized through coordination with either four (tetrahedral interstitial sites) or six (octahedral lattice sites) oxygen atoms (Fig. 2D) (*31*, *48*). To assess which coordination environment is more favorable for the lithium ion (i.e., within the SSE framework or the hydration shell in solution), leaching experiments with the SSE were performed (fig. S9). While the dissolution of lithium ions from the SSE is entropically favorable, no lithium ions were found to leach into solution over several days. Therefore, it can be implied that the SSE framework provides superior stabilization of the lithium ion (i.e., enthalpic favorability) compared to the hydration shell in solution.

The suspected offset of the dehydration energy penalty is further validated by the magnitude of the measured enthalpic barrier closely agreeing with the activation energy values commonly reported for similar NASICON-like structures in the solid-state battery literature (*27*, *48*). Accordingly, we find that the enthalpic barrier of the SSE, both in aqueous and solid-state applications, primarily reflects the energy penalty incurred for defect formation and migration. Specifically, partitioning of an ion into the SSE framework simultaneously requires the formation of either a vacancy or interstitial defect through energetically unfavorable bond breakage or formation, effectively contributing to the overall enthalpic energy barrier. Additionally, subsequent migration of the lithium ion through the crystalline framework further adds to the enthalpic barrier, as lithium ions cross from highly stabilized lattice sites through relatively less stable interstitial sites (*25*, *27*).

Similarly, the relatively large enthalpic barrier of the CEM may be interpreted through consideration of the ion interactions with the negative fixed-charge groups of the membrane (i.e., sulfonate groups on the polymer backbone). While the negative fixed-charge groups in the CEM provide favorable electrostatic interactions with lithium ions for partitioning, once inside the CEM, such interactions effectively hinder transport and must be overcome for the ion to traverse the membrane. Furthermore, it is important to note that in contrast to the closely packed and highly ordered structure of the SSE, the negative fixed-charge groups in the CEM are widely spaced (on the order of 10 Å) and randomly dispersed (*49*, *50*). Thus, during both the ion partitioning and transmembrane diffusion steps, the lithium ion is expected to pass through higher energy transition states in the case of the CEM as compared to the SSE, in which oxygen atoms are ideally arranged for the stabilization of dehydrating (i.e., partitioning) and migrating lithium ions.

While migrating through either the SSE or CEM ultimately incurs an enthalpic penalty, the entropic changes in each membrane type provide alternate contributions to the total free energy barrier. Specifically, ion transport in the SSE is found to result in an overall decrease in entropy compared to bulk solution, whereas the entropy change in the CEM is found to be positive (i.e., negative −*T*Δ*S*), in agreement with entropy barriers reported for cation transport in CEMs (*49*). Accordingly, transport in the CEM is found to be entropically favorable, effectively compensating for the corresponding high enthalpic barrier. In contrast, transitioning from bulk solution to the lower entropy states in the SSE requires energetic input, thus adding to the enthalpic barrier.

Transport of an ion through a membrane inherently confines the mobility of the ion compared to bulk solution, thus decreasing entropy. However, the nanometer-scale pores in ion-exchange membranes are large and facilitate relatively unhindered freedom of molecular motion compared to other membrane types (*38*, *51*). Interactions with the polymer chains and fixed-charge groups, nonetheless, are likely to lead to disruption of the hydration shell around the lithium ions (Fig. 2C). Such temporary breakage and rearrangement of the structurally ordered hydration shell (i.e., in the activated state) would effectively increase the entropy of the lithium ion, which is expected to culminate in a net gain in entropy during ion migration through the CEM. In contrast to a CEM, the freedom of motion of lithium ions is severely more restricted in an SSE, with the ions only being able to occupy and migrate through the lattice (octahedral) and interstitial (tetrahedral) sites of the highly ordered and rigid crystalline network (Fig. 2D). Furthermore, transport through the SSE requires migration through size-restrictive bottlenecks as the lithium ions hop from lattice to interstitial sites. For NASICON-like SSE materials, in particular, the size of these migration bottlenecks is only a few angstroms (*52*–*54*); thus, traversing such channels necessitates single-file ion transport and poses substantial steric hindrance effects. Such confined and ordered transport increases entropy relative to that of the hydrated lithium ion in bulk solution, as supported by the substantial entropic barrier measured for the SSE.

### Approaching perfect selectivity for lithium transport in SSE

Our assessment of the SSE, thus far, has focused on understanding the mechanisms of lithium ion and water transport in the material while highlighting how such phenomena fundamentally differ from that in more conventional nanoporous membranes. Hence, until now, only single-salt solutions consisting of lithium chloride were used. However, in practical aqueous membrane applications, such as lithium extraction, it is necessary to separate lithium ions from a complex mixture containing relatively high concentrations of coexisting ions. Specifically, most relevant source waters contain substantial concentrations of sodium and magnesium ions (*9*, *11*), making lithium extraction with membranes highly challenging. Accordingly, we assessed the selectivity of the SSE for lithium transport against both sodium and magnesium.

**Fig. 3.**
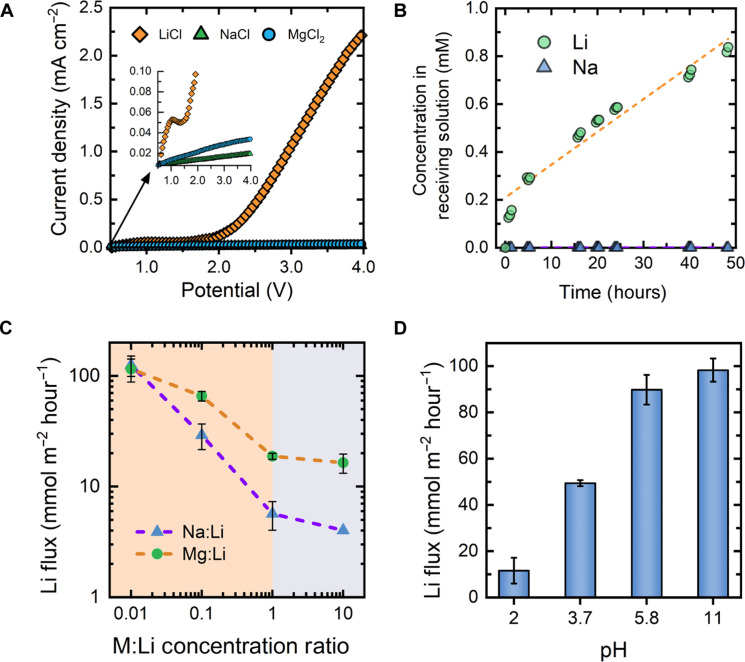
Assessment of ion-ion selectivity in the SSE. (**A**) The current response from linearly sweeping the potential at a scan rate of 2 mV s^−1^. A 100 mM single-salt solution of either lithium chloride (orange diamonds), sodium chloride (green triangles), or magnesium chloride (blue circles) was used and continuously recirculated through both the feed and receiving channels. The inset shows the current density on a truncated scale to more clearly show the data and trends in the low-current regime. (**B**) The lithium (green circles) and sodium (blue triangles) concentrations in the receiving solution over a 50-hour long-term ion competition experiment. A multi-salt feed solution of 10 mM LiCl and 10 mM NaCl was continuously supplied to the feed channel over the experiment duration, while the receiving solution was initially 10 mM KCl. The orange dashed line shows the linear fit of the lithium concentration data. (**C**) Multi-salt ion competition experiments in which the feed solution consisted of varying molar ratios of either Na:Li (blue triangles) or Mg:Li (green circles). The lithium flux for each of the experiments is shown, while the sodium and magnesium fluxes remained undetectable across all experimental conditions. (**D**) The effect of feed solution pH on the lithium flux. The pH of a 10 mM LiCl solution was adjusted by dosing either hydrochloric acid or lithium hydroxide.

To begin our assessment of selectivity, we performed single-salt experiments consisting of 100 mM solution of either lithium chloride, sodium chloride, or magnesium chloride. As the applied potential was linearly increased, the current response, which is reflective of the transmembrane ion flux, was monitored (Fig. 3A). With the lithium chloride solution, the current remains near zero and rapidly encounters a plateau region until reaching a potential of ~1.5 V, at which an inflection point is observed. Notably, ~1.5 V is the practical water splitting potential, suggesting that, at lower applied potentials, the lithium flux across the SSE is limited by the occurrence of electrochemical reactions at the electrodes. Nonetheless, beyond 1.5 V, the current rapidly grows, indicating that higher applied potential leads to greater flux of lithium ions across the SSE, as would be expected.

Upon replacing the lithium chloride solution with sodium chloride or magnesium chloride, however, the profile of the current response is entirely changed and the magnitude of the current markedly drops. Specifically, for both the sodium and magnesium solutions, the current shows a relatively linear increase over the entire applied potential window (Fig. 3A, inset graph), with no sign of an onset potential as was observed with the lithium chloride solution. Such a result suggests that, with both the sodium chloride and magnesium chloride solutions, there is a lack of viable charge carriers across the SSE and that the marginal current observed can likely be attributed to unavoidable leakage current in the electrochemical cell, rather than transmembrane ion flux. Notably, toward higher applied potentials, where the current response for the lithium chloride solution is substantial, the current diverges from that of sodium and magnesium chloride solutions by nearly two orders of magnitude, alluding to potentially high lithium selectivity.

While the single-salt experiments provide an initial indication of promising ion-ion selectivity in the SSE, competitive ion-membrane interactions can lead to considerable variation in multi-salt solutions (*55*). Hence, we continued our evaluation of lithium selectivity through a long-term ED experiment, in which a solution consisting of equimolar lithium and sodium (10 mM each) was continuously fed to the cell. Over the duration of the 2-day constant voltage experiment, we found that the flux of lithium remained fairly constant after the first hour, leading to a linear increase in the lithium concentration in the receiving solution over time (Fig. 3B). Accordingly, the SSE is found to have a high degree of stability under electrodialytic operation and in the presence of moderate concentrations of sodium. Throughout the entirety of the long-term experiment, there was no detectable sodium flux, as confirmed by both ion-chromatography and inductively coupled plasma mass spectrometry (ICP-MS). Hence, the SSE is found to provide near-perfect selectivity for lithium transport over sodium.

To further investigate the selectivity of the SSE and uncover potential limitations, we systematically tested performance with various feed solution mixtures and concentrations. Specifically, we performed mixed-salt experiments in which 10 mM LiCl was combined with varying concentrations of either sodium or magnesium chloride, effectively covering a wide range of molar ratios between the competing cation and lithium ion. We note that a concentration of 10 mM LiCl was used throughout the competitive ion transport tests to reflect the minimum lithium ion concentration required in practical feedwaters for economically viable extraction (*7*).

Across all the solution combinations investigated, no sodium or magnesium flux was detected (by either ion-chromatography or ICP-MS), resulting in ideal selectivity for lithium transport. Nonetheless, while the selectivity was maintained across all solution conditions investigated, a critical trade-off relationship between the lithium flux and the competing ion concentration was revealed (Fig. 3C). Particularly, at low competing ion concentration (i.e., M:Li molar ratio of 0.01), the lithium flux for both the sodium and magnesium mixed-salt solutions remains comparable to that observed with pure 10 mM LiCl solution. However, as the molar ratio of the competing cation is progressively increased to 0.1 and 1.0, substantial decline in the lithium flux is observed, indicating that the competing ions, despite not crossing the SSE, pose considerable hindrance for lithium permeation. Such an effect may suggest that competing cations inhibit the partitioning of lithium ions into the SSE via surface site blocking, with further discussion of this hypothesized mechanism provided in the following section. Notably, as the competing ion concentration ratio is further increased from 1.0 to 10.0, the relative decline in the lithium flux (for both sodium and magnesium containing solutions) begins to plateau, implying that the surface sites on the SSE begin to saturate with the competing ion.

The potential interference from protons in solution was also investigated by determining the lithium flux through the SSE with feed solutions at various pH (Fig. 3D). At circumneutral and high-pH conditions, in which the proton concentration is negligible, the lithium flux was found to remain relatively unchanged. Although a slight increase in lithium flux is observed at pH 11, we attribute this to the increased lithium concentration in the feed solution, a consequence of raising the pH through dosing of lithium hydroxide, as depicted in fig. S10. It should be noted that, while a high pH provides a substantial concentration of hydroxide ions, which are small and highly mobile compared to the lithium ion, under an applied electric field, the hydroxide ions migrate away from the SSE, effectively minimizing impact on the flux of lithium. In contrast, with lower pH feed solutions, the flux of lithium was found to be severely reduced, implying that protons, like other cations, pose competitive effects with lithium. Notably, our results show that, even at a H^+^:Li^+^ ratio of 0.01 (i.e., pH 4), the lithium flux drops by nearly half, while, at the same competing ion ratio, sodium and magnesium ions had no impact on the lithium flux. Thus, lithium transport across the SSE is found to be more sensitive to the presence of protons as compared to salt ions.

To determine whether protons traverse the SSE, the pH of the receiving solution was monitored over the duration of the pH 2 and pH 3.7 experiments. As seen in fig. S11, the experiments conducted at pH 3.7 unintuitively showed an increase in the pH of the receiving solution, rather than a decrease in pH which would indicate transmembrane proton transport. However, this result may be understood by considering that the electrochemical reduction reactions at the cathode (e.g., hydrogen evolution reaction) generate hydroxide ions, which can readily migrate across the anion exchange membrane (AEM) that separates the electrode rinse solution from the receiving solution (Fig. 1B). In effect, the pH change from potential proton migration across the SSE is likely to be masked in the case of the pH 3.7 experiments. Nonetheless, for the experiments conducted at pH 2, a decline in the receiving solution pH was observed over time, indicating a clear flux of protons through the SSE that outweighed the corresponding flux of hydroxide ions through the AEM. Such a result demonstrates that the reduction in lithium flux at low pH values is likely due to the competitive migration of protons throughout the SSE, unlike in the case of salt ions that also reduce the lithium flux, but without permeating the membrane.

### Underlying mechanisms of ion selectivity in the SSE

Through our assessment of the SSE, we demonstrated unprecedented selectivity for lithium transport over both sodium and magnesium. Furthermore, a unique relationship was uncovered, whereby the lithium flux was found to decline with increasing concentration of coexisting sodium and magnesium ions, although no transmembrane flux of the competing ions was observed. While such performance is highly promising in the context of ion separations, the observed phenomena are not common of other ion-selective membrane processes. Hence, in this section, we combine molecular simulations with experimental characterizations to elucidate the unique underlying mechanisms governing competitive ion transport in the SSE.

To gain insight into the interactions between the ions and SSE, molecular dynamics simulations were performed on three systems that closely resembled our experimental conditions. Specifically, an electric field was applied across an SSE separating two aqueous salt solutions. The receiving solution remained potassium chloride, while the feed solution was systematically varied to contain either (i) pure lithium chloride, (ii) equimolar lithium and sodium chloride, or (iii) equimolar lithium and magnesium chloride. We note that, although the commercial SSE material used in this study is doped (i.e., a portion of the titanium atoms are substituted with germanium, silicon, and aluminum) to enhance ionic conductivity, for our general purpose of understanding competitive ion effects, we simulated the SSE using the simplified crystal structure of unsubstituted yet structurally similar LiTi_2_(PO_4_)_3_.

**Fig. 4.**
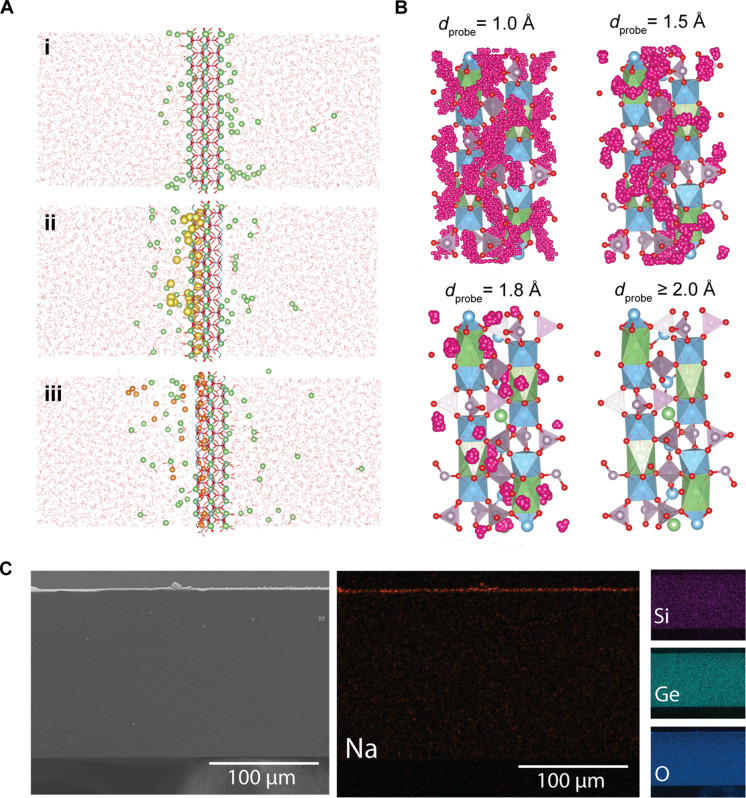
Elucidating the mechanisms of ion-ion selectivity in the SSE. (**A**) Molecular dynamics snapshots captured at the end of the simulation period for each of the three systems analyzed. In each simulation box, a LiTi_2_(PO_4_)_3_ membrane was placed between two aqueous solutions. The simulations were initialized with the right-hand side solution containing potassium chloride (not shown), while the solution on the left-hand side contained lithium and competing ions. Each system initially contained the same number of lithium ions (green spheres) in the left-hand solution. In system [(A), i], only lithium chloride was in the left-hand solution, whereas, in [(A), ii] and [(A), iii], the number of lithium ions was matched by an equal amount of sodium ions (yellow spheres) and magnesium ions (orange spheres), respectively. (**B**) Visualization of PoreBlazer simulations performed with various diameter spherical probes (*d*_probe_). The LiTi_2_(PO_4_)_3_ unit cell is overlaid with pink spheres that indicate accessible positions for the probe. The color coding of the LiTi_2_(PO_4_)_3_ lattice is the same as that shown in Fig. 1A. (**C**) Scanning electron microscopy–energy dispersive x-ray spectroscopy (SEM-EDS) images of the SSE cross section after use in a 48-hour experiment with 10 mM NaCl and 10 mM LiCl feedwater. Elemental maps of sodium (red), silicon (purple), germanium (teal), and oxygen (blue) are shown in separate panels.

As shown by the simulation snapshots for each system (Fig. 4A), lithium ions were found to traverse the SSE under all conditions. Furthermore, the molecular dynamics simulations reinforce our water transport experiments, showing that water molecules are unable to penetrate the SSE structure (fig. S12), and, thus, lithium ions migrate across the SSE in an anhydrous state (fig. S13). Unlike lithium, simulations show that sodium and magnesium ions are unable to cross the SSE structure, in agreement with our experimental results. Notably, the simulations show a buildup of sodium and magnesium ions at the surface of the SSE, with neither ion being capable of penetrating past ~3 Å of the SSE’s top atomic layer. The magnesium ions, nonetheless, are found to distribute more evenly within this surface region as compared to sodium ions, which is likely attributed to their smaller ionic size (fig. S14).

The impermeability of the sodium ion through the SSE may be attributed to its relatively large ionic size compared to lithium ions. As previously discussed, transport in the SSE occurs under anhydrous conditions through a crystalline lattice; thus, the crystallographic radii of the ions must be considered. Specifically, for coordination numbers ranging from four to six (as would typically be encountered in the structure of the SSE), the lithium ionic diameter ranges from 1.18 to 1.52 Å, whereas sodium ranges from 1.98 to 2.04 Å (*56*). While such sub-angstrom ionic size differences cannot readily be differentiated in conventional nanoporous membranes, the lithium conducting NASICON materials have been shown to have angstrom-scale conducting channels (*52*–*54*). Thus, such materials could effectively provide size sieving of the larger sodium ions while facilitating the passage of the relatively small lithium ions.

To further validate and visualize this size exclusion mechanism, we performed additional simulations using PoreBlazer (*36*). As shown in Fig. 4B, variously sized spherical probes were administered to effectively map out the size of the interstitial sites within the SSE structure. When a probe size of 1.0 Å was applied, most of the interstitial volume was accessible, as indicated by the pervasiveness of the pink spheres. However, as the probe size was gradually increased, the accessibility of the probe to the interstitial spaces was markedly reduced (fig. S15). Notably, with a probe size of 1.5 Å, which may be considered representative of the size of a lithium ion, the probe accessible fractional free volume shrinks from 62 to 29%. Nonetheless, as can be seen in Fig. 4B, the interstitial spaces between the lattice sites of lithium (i.e., shown as the green octahedrons) remain occupiable, allowing for lithium ions to still pass from one lattice site to another. When the probe size is increased to 1.8 Å, however, this interconnectedness of lattice sites is broken, and further increasing the probe size to 2.0 Å, the size of a sodium ion, leads to complete loss of accessibility to the free volume elements. Hence, the effective bottleneck size for ion migration determined by our simulations is between 1.8 and 2.0 Å, in good agreement with values previously reported through more standard geometric calculations (*52*). Accordingly, we reason that sodium ions, when provided enough energy, may exchange for lithium ions in the lattice sites; however, further penetration into the SSE framework is likely prevented by their inaccessibility into the size restrictive interstitial sites.

While sodium ions are excluded from the SSE because of a size-sieving mechanism, magnesium ions, which effectively are the same diameter as lithium ions (table S3) (*56*), are also found to be impermeable. Such a result implies that ion selectivity in the SSE not only is dependent on the ionic size but also is related to the charge of the ion. Specifically, the divalent nature of magnesium ion is expected to substantially alter its interaction with the SSE framework, which is composed of mobile monovalent lithium ions. Whereas monovalent ions, like sodium, may exchange for the lithium ions in the SSE structure, while still maintaining overall charge neutrality in the framework, the introduction of a magnesium ion into the framework would inherently require the formation of an additional vacancy defect. Thus, the (enthalpic) energy barrier for divalent ion migration through the SSE is expected to be considerably larger than that of monovalent ions, as supported by recent density functional theory calculations (*57*). Furthermore, the divalent nature and small ionic size of magnesium lead to a larger and more tightly held hydration shell, relative to monovalent sodium and lithium ions (table S3 and fig. S16). Considering both the large hydration energy of magnesium ions and the incompatibility of divalent ions (in place of lithium ions) in the SSE structure, partitioning of magnesium ions into the SSE is likely to incur a large energetic penalty (i.e., the energy barrier of magnesium ion dehydration is not offset by stabilization within the SSE framework).

In addition to the exceptional ion selectivity, our competitive ion transport experiments also revealed an unexpected relationship between the lithium flux and the competing ion concentration. Particularly, although zero flux of sodium or magnesium was observed, the lithium flux was severely reduced in their presence. Notably, our molecular dynamics simulations show a similar trend, whereby the lithium flux was reduced when sodium and magnesium ions were introduced to the system, albeit to a smaller extent. As suggested by the simulations shown in Fig. 4A, this flux reduction effect is likely attributed to the accumulation of the impermeable sodium and magnesium ions near the SSE surface. Specifically, while the competing ions do not cross the SSE, their surface level ion-exchange into the SSE effectively blocks lithium ions from accessing otherwise viable lattice and interstitial sites for partitioning. Both our experimental (Fig. 3C) and simulation (Fig. 4A) results showed a more marked reduction in the lithium flux when the interfering ion was sodium as opposed to magnesium. Hence, it may be inferred that, compared to sodium ions, magnesium ions interact less with the SSE structure or are more readily reversibly exchanged with lithium ions from the feed solution.

To further support our theory that interfacial ion-exchange hinders the flux of lithium, we performed SEM-EDS mapping on the cross section of the SSE after a long-term experiment where the feed solution consisted of equimolar lithium and sodium (Fig. 4C). The elemental mapping of the cross section clearly shows homogenous distribution of oxygen, silica, and germanium across the entire membrane thickness, as would be expected from the chemical structure of the SSE. Notably, sodium was also detected, although it was found to only be present at the feed-side surface of the material. As the surface of the SSE had been thoroughly rinsed with deionized water to remove surface adsorbed species before imaging, this result provides strong evidence that sodium integrates into the structure of the SSE but is unable to migrate past the surface layer, thereby hindering the transport of lithium ions. Furthermore, we performed x-ray diffraction on the SSE both before and after the long-term mixed-salt experiment. As shown in fig. S17, the diffraction pattern of the SSE showed notable deviation from the pristine after the ED test, indicating alteration of the crystalline structure. Upon further analysis of the phases, we identify that the changes in the diffraction pattern reflect transformation of Li_4_P_2_O_7_ to Na_4_P_2_O_7_, supporting that sodium exchanges for lithium at the solution-SSE interface.

## DISCUSSION

Over recent decades, SSE materials have attracted considerable research attention, being primarily guided by application in battery technologies (*25*). In this study, we demonstrated that SSEs could also be highly promising as ion-selective membranes for aqueous separations. By systematically comparing a state-of-the-art NASICON-like lithium ion conductor to a cation-exchange membrane, we demonstrated how water and lithium transport fundamentally vary in an SSE as compared to conventional polymeric membranes. We revealed that, unlike other classes of membrane materials, ion transport in SSE frameworks occurs under anhydrous conditions via solid-state diffusion mechanisms, ultimately limiting the attainable ion flux. Nonetheless, while the lithium ion permeability of the SSE was determined to be lower than that of traditional membranes, the highly ordered structure and angstrom-scale migration pathways provide unparalleled selectivity for the transport of lithium over both sodium and magnesium ions.

**Fig. 5.**
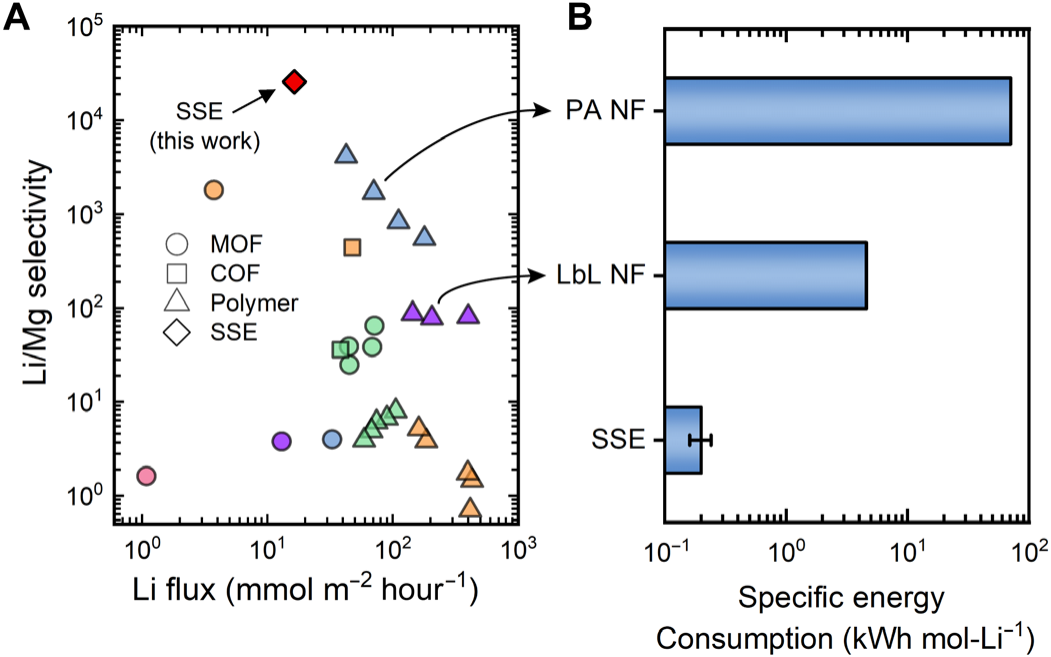
Comparison of the SSE to other reported lithium ion selective membranes. (**A**) The lithium-magnesium selectivity ratio and the lithium ion flux for various membranes in the literature. A different symbol is used to indicate the various classes of membrane materials: pure polymer membranes, and membranes that incorporate either metal-organic frameworks (MOFs) or covalent organic frameworks (COFs). For each membrane type, a distinct color represents data from a different reference. All data shown is also provided in table S4. The reported performance for the SSE corresponds to the experiments performed with a Mg:Li molar ratio of 10:1. (**B**) The specific energy consumption (SEC) of lithium extraction using the SSE as compared to pressure-driven nanofiltration membranes. The SEC values for the layer-by-layer (LbL) NF membrane (*68*) and highly selective polyamide (PA) NF membrane (*16*) were calculated on the basis of the conditions reported in the respective works. For each of the SEC values, the feed solution is a binary mixture containing approximately a 10:1 molar ratio of Mg:Li. Arrows are provided to indicate the points in (A), which correspond to the evaluated NF membranes in (B).

Although the lithium flux through the SSE is severely reduced in the presence of competing cations, the flux values still remain comparable to those observed in considerably less selective membrane materials, such as polyelectrolyte multilayer films (*58*, *59*), metal-organic frameworks (*19*, *23*, *24*, *60*–*62*), and covalent organic frameworks (*22*, *63*). To highlight the relative performance of the SSE, we show the lithium-magnesium selectivity as a function of the lithium flux for several membranes reported in the literature (Fig. 5A). To compare the results of our study to the literature, we approximated a lithium-magnesium selectivity value using the limit of detection of the ICP-MS (fig. S18). However, it is important to note that no magnesium flux was detected in our experiments, and, thus, the reported selectivity is a minimum value based on the experimental measurement capabilities. While Fig. 5A shows that most materials, including the SSE, abide by a general permeability-selectivity trade-off relationship, the order-of-magnitude gain in selectivity offered by the SSE comes at a relatively small expense in flux. Furthermore, we note that the reported lithium flux of the SSE is not optimized and can potentially be increased by varying the operating conditions (e.g., stronger applied electric field or higher fluid velocity to reduce concentration polarization effects) or by developing thinner membranes.

It is also important to note that, in Fig. 5A, only the lithium-magnesium separation performance was considered because of the relative abundance of such data in the literature. However, in practical source waters, the concentration of lithium ions is generally dwarfed by that of coexisting sodium ions (*9*, *64*). While solubility differences between sodium and lithium precipitates (e.g., Na_2_CO_3_ and Li_2_CO_3_) may be exploited to achieve downstream chemical separation, the presence of high sodium concentrations can compromise final lithium product purity and can adversely affect the efficiency of the DLE process (*9*, *64*–*67*). In electro-driven membrane processes, in particular, the energy consumption directly scales with the current (i.e., number of ions transported). Thus, high lithium-sodium selectivity is critical to minimize the amount of current wasted toward the transport of sodium ions. Nonetheless, reported materials that show high lithium-magnesium selectivity typically lack sufficient selectivity between lithium and sodium ions, limiting their practical viability (*18*, *19*, *21*, *22*).

Similarly, reports on lithium-selective membranes commonly overlook the importance of lithium-water selectivity. Although pressure-driven nanofiltration (NF) membranes have demonstrated high lithium-magnesium selectivity with relatively high lithium flux, it is critical to realize that most of the energy input in such systems is expended toward the transport of water molecules, rather than lithium ions. Hence, the specific energy consumption (SEC) for pressure-driven lithium extraction (details provided in the Supplementary Materials), using even the most promising NF membranes reported in the literature (*16*, *68*), remains orders of magnitude higher than that of the SSE (Fig. 5B). Notably, the SEC of lithium extraction using the SSE may be even further reduced by increasing the lithium ion flux (e.g., with thinner membrane design) or by scaling up of the demonstrated system to a multicell pair ED stack, in which the contribution of the redox potential of the electrodes becomes negligible (*69*). While the low SEC of SSE ED suggests that the process may be highly promising for practical lithium extraction, a comprehensive cost analysis that also considers capital expenditure is necessary to draw conclusions on potential economic competitiveness.

Overall, this study highlights the highly promising application of SSEs to lithium recovery while also providing fundamental insights into ion transport when such materials are used in aqueous systems. Nonetheless, we note that further work is required to demonstrate the effectiveness of the SSE when applied to increasingly complex and saline source waters. Particularly, while ideal lithium selectivity was observed throughout the conditions used in this study, which encompass the concentrations encountered in many aqueous lithium sources, markedly increasing the concentration of competing ions or the applied voltage could eventually provide sufficient electrochemical potential to facilitate transmembrane transport of the competing ions. We speculate that, in such a case, the SSE material would effectively become doped with the competing ion, fundamentally changing its atomic composition and transport properties. Hence, future studies will focus on identifying such doping limits and how they relate to practical electrodialytic operation.

The extension of SSE materials into aqueous ion separations presents both new research challenges and opportunities. In this study, we used only one NASICON-like SSE as a model material; however, systematic evaluation of various classes of SSE materials (e.g., LISICON, garnet, and perovskite) may enable further understanding of the underlying mechanisms and potential of solid-state ion conductors in the context of ion-ion selectivity. Nonetheless, such investigations would require the development of more water-stable SSE materials, which are now relatively scarce (*25*, *32*). Additionally, applying the SSE to an aqueous system, rather than a solid-state battery, inherently alters the interfacial phenomena. Although substantial research focus has been directed toward the understanding and optimization of the SSE-electrode interface in solid-state batteries, the SSE-solution interface in ED introduces unique considerations. For example, while the SSE-solution interface obviates the common concern of lithium metal dendrite growth in solid-state batteries, the electrochemical stability window of the SSE and water at the interface must be thoroughly evaluated to avoid potential material degradation. Future study of the SSE-solution interface is also particularly critical for developing strategies to mitigate the blockage of the SSE surface sites by competing ions, thus allowing for a high lithium flux to be maintained in the presence of coexisting cations.

With supply shortages rapidly approaching, the need to harness lithium from increasingly complex aqueous ionic mixtures continues to grow. On the basis of the exceptional lithium ion selectivity demonstrated, we expect that SSE materials will be at the forefront of DLE technologies. However, we note that the potential application of SSE membranes is not limited to lithium alone. Although focus on battery technologies has culminated in an assortment of lithium and sodium ion conductors, the extreme selectivity demonstrated by solid-state transport mechanisms is expected to inspire the development of alternate ion conductors for the efficient extraction of other critical elements from aqueous sources.

## MATERIALS AND METHODS

### Materials and chemicals

Lithium chloride (J.T. Baker, >99.5%), sodium chloride (Sigma-Aldrich, >99%), potassium chloride (Sigma-Aldrich, >99%), magnesium chloride hexahydrate (Sigma-Aldrich, >99%), and ammonium chloride (J.T. Baker, >99.5%) were dissolved in MilliQ ultrapure deionized water (>18 megaohms·cm) for the preparation of various salt solutions. A commercially available NASICON-type solid-state electrolyte (Ohara LICGC AG-01) was used throughout the study and replaced after each experimental set or as needed (i.e., drop in ionic conductivity or visible cracking of the material was observed). Notably, this particular SSE was selected because of its high lithium ion conductivity and exceptional water stability, as reported by the manufacturer. A commercial cation-exchange membrane (Fumasep FKD-PK-75) and an anion-exchange membrane (Fumasep FAS-PET-130) were used. A CH Instruments 600E potentiostat was used to perform all electrochemical techniques throughout the study.

### Determination of ion flux and selectivity

The ion flux across the SSE and the CEM was determined by operating a custom-built ED cell (Fig. 1A and fig. S2), with details on the cell design provided in the Supplementary Materials. The ED system was operated in batch mode, in which 20 ml of the feed solution (varying composition depending on the experiment) and 20 ml of the receiving solution (10 mM KCl) were continuously recirculated at a flow rate of 1 ml min^−1^ through the corresponding channels in the cell (Fig. 1B). For experiments in which the pH of the feed solution was varied, lithium hydroxide or hydrochloric acid was used to increase or decrease the pH, respectively. Potassium chloride was used in the receiving solution, as opposed to deionized water, to provide solution conductivity. A 10 mM solution of Na_2_SO_4_ was used as the electrode rinse solution, except when sodium was present in the feed solution, in which case the electrode rinse solution was substituted with 10 mM MgSO_4_ (i.e., to avoid any potential error in the cation flux measurement stemming from co-ion leakage across the anion-exchange membranes). The electrode rinse solution (150 ml) was continuously recirculated at a flow rate of 8 ml min^−1^ (to sweep away generated gases from water splitting reactions), and the rinse solutions from both the anode and cathode were mixed into the same batch, ensuring minimal pH variation of the bulk electrode rinse solution over the duration of the batch.

Before beginning the experiment, the ion-exchange membranes were soaked in the corresponding solution overnight for equilibration. Upon assembling the system, deionized water was pumped through each of the chambers in a single-pass operation mode (i.e., disposing of the effluent after a single pass through the cell) for 20 min to remove any adsorbed ions on the surface of the membranes. Air was then pumped through the channels to empty the chambers of any residual water, after which the experimental solutions were recirculated through their corresponding channels for 30 min to bring the system to an equilibrium.

A constant potential of 4 V was applied over a duration of 2 hours, and the current response was recorded in 1-s intervals. The feed and receiving solutions were sampled every 20 min, and sodium, lithium, and magnesium concentrations were measured using a Metrohm 940 Professional IC Vario ion chromatograph as well as a PerkinElmer Nexion 5000 multi-quadrupole ICP-MS. The flux of each species (*J*_*i*_) was calculated according to

$$J_{i}=\frac{∆C_{i}V}{A_{m}∆t}$$ (1)

where ∆*C*_*i*_ is the change in the concentration of the species in the receiving solution, *V* is the volume of the recirculating receiving solution, *A*_m_ is the exposed membrane area (3.2 cm^2^), and ∆*t* is the time duration over which the concentration change is measured. We note that the ion flux measurements were determined according to the concentration data collected from 60 min onward, a period over which the flux (i.e., current) had reached a steady state value. All experiments that measured membrane flux were carried out in triplicate.

A long-term experiment with the SSE was conducted for 50 hours, over which a constant potential of 4 V was applied. A feed solution consisting of 10 mM LiCl and 10 mM NaCl was continuously fed to the feed chamber at a rate of 1 ml min^−1^ in single-pass operation (i.e., the effluent was not recirculated but rather disposed). A 20-ml solution of 10 mM KCl was recirculated through the receiving chamber over the entire duration of the experiment, and 0.1 ml samples were taken from this vessel. The electrode rinse solution (2 liters of 10 mM MgSO_4_) was recirculated at a rate of 8 ml min^−1^. After the long-term experiment, the SSE was removed from the cell, thoroughly rinsed with ultrapure deionized water and characterized.

The selectivity for lithium transport over other cations (*S*_Li/*i*_) is determined for multi-salt feed solutions according to

$$S_{\text{Li}/i}=\frac{∆C_{\text{Li}}C_{f,i}}{∆C_{i}C_{f,\text{Li}}}$$ (2)

where $∆C_{\text{Li}}$ is the change in the lithium concentration in the receiving solution over the duration of the batch, while $C_{f,\text{Li}}$ and $C_{f,i}$ are the feed concentrations of lithium and the competing species, respectively.

Linear sweep voltammetry experiments were conducted using a single-salt solution (i.e., 100 mM NaCl, LiCl, or MgCl_2_) which was fed to both the feed and receiving channels. The solution was recirculated into the same reservoir, ensuring minimal variation in concentration over the duration of the experiment. The electrode rinse solution was 10 mM Na_2_SO_4_ or MgSO_4_, depending on the feed solution used. The voltage was swept at a rate of 2 mV s^−1^ from 0 V to a final potential of 4 V.

### Measurement of membrane conductivity and energy barriers

Measurements of the membrane conductivity and energy barriers were obtained using a modified version of the ED cell, where the central serpentine flow channels were replaced with 1.5-inch (3.81-cm)–thick open flow channels (fig. S6). Luggin capillaries were inserted into the flow channels and filled with 1 M KCl, and an Ag/AgCl reference electrode (Pine Research LowProfile) was immersed in the solution of each capillary. The Luggin capillary tips were in close proximity to the central membrane (i.e., the membrane under investigation), and the potential difference across the tips was measured via the reference electrodes using a digital multimeter.

The same solution of 0.5 M LiCl (250 ml) was recirculated through each of the inner flow channels; hence, despite lithium transporting across the central membrane (i.e., from the feed to the receiving chamber) under an applied current, the overall lithium concentration in the feed solution remained constant over the duration of the experiment, effectively minimizing temporal variation in the measured potential difference. The flow rate through the central chambers was set to 20 ml min^−1^, and stirring was provided in each channel with a magnetic stir bar to ensure sufficient mixing and minimization of boundary layer effects. A 500-ml solution of 10 mM Na_2_SO_4_ was recirculated through both of the electrode rinse channels at a flow rate of 15 ml min^−1^.

The conductivity of the central membrane was determined by measuring the potential difference across the membrane (∆ϕ_m_) at various applied current densities (i.e., 0.2, 0.4, 0.6, 0.8, 1, and 1.2 mA cm^−2^). The slope of the measured membrane potential drop versus the applied current density gives the resistance (*R*_m_) of the membrane (fig. S7). The membrane conductivity (σ_m_) can then be calculated as

$$\sigma_{m}=\frac{\delta_{m}}{R_{m}A_{m}}$$ (3)

where δ_m_ is the thickness of the membrane (75 μm for the CEM and 150 μm for the SSE) and *A*_m_ is the exposed membrane area (6.45 cm^2^). Measurement of membrane conductivity was conducted in duplicates.

The potential difference measured across the capillaries (∆ϕ_tot_) includes the potential drop across the membrane as well as the potential drop across the solution layers (i.e., the solution between the capillary tips and the membrane surface). Hence, for the purpose of determining the membrane conductivity, the solution potential drop (∆ϕ_sol_) must be subtracted from the total measured potential difference. The solution potential drop was determined in each experiment by continuously (in 20-s intervals) measuring the conductivity in the feed solution and assuming that the measured conductivity is equal to that of the solution between the capillary tips and membrane. Thus, the potential difference across the membrane can be determined as

$$∆\phi_{m}=∆\phi_{\text{tot}}-∆\phi_{\text{sol}}=∆\phi_{\text{tot}}-\frac{d_{\text{tips}}i}{\sigma_{\text{sol}}}$$ (4)

where *i* is the applied current density and *d*_tips_ is the distance between the tips of the capillaries. We determined the distance between the capillary tips to be 2.1 mm by measuring the potential difference across the reference electrodes without the inclusion of the central membrane (i.e., ∆ϕ_sol_ = ∆ϕ_tot_) at a fixed current density of 1 mA cm^−2^.

The energy barrier for lithium transport across the central membrane was determined by assessing the temperature dependence of the membrane conductivity (i.e., permeability). Specifically, the membrane conductivity was measured at 25°, 30°, 35°, and 40°C, where the temperature was controlled using a continuously exchanged (i.e., recirculating) water bath. The bath temperature was maintained using a Cole Parmer water heater/circulator, while the temperatures in the bath and the lithium chloride feed solution were monitored using a thermometer and Oakton 4000 conductivity/temperature probe, respectively. To avoid heat loss during pumping of the solutions, the cell was submerged in the water bath up to the height of the titanium rods (i.e., the potentiostat lead connections). Each temperature was maintained for >30 min, and measurements of the potential difference and solution conductivity were taken in 10-min intervals. The average of the four collected data points at each temperature were used as the membrane conductivity. Each of the energy barrier experiments were performed in duplicates.

According to transition state theory, the free energy barrier (Δ*G*), the enthalpic energy barrier (Δ*H*), and the change in entropy (Δ*S*) associated with ion transport across a membrane are related to the membrane permeability (*P*) by (*41*, *42*)

$$P=\frac{{\lambda_{i}}^{2}k_{B}T}{h\;\delta_{m}}e^{-\Delta G/\mathrm{RT}}=\frac{{\lambda_{i}}^{2}k_{B}T}{h\;\delta_{m}}e^{\Delta S/R}e^{-\Delta H/\mathrm{RT}}$$ (5)

where λ is the molecular jump length, *k*_B_ is the Boltzmann constant, *T* is the absolute temperature, *h* is Planck’s constant, *R* is the ideal gas constant, and the subscript *i* refers to either the SSE or CEM.

For electro-driven ion transport, the membrane permeability can be expressed in terms of the membrane conductivity according to (*44*)

$$P=\frac{\mathrm{RT}\sigma_{m}}{F^{2}C_{i}\delta_{m}}$$ (6)

where *F* is Faraday’s constant and *C* is the ion concentration in the membrane. By substituting Eq. 6 into Eq. 5, the energy barriers can be directly related to the temperature dependence of the membrane conductivity. Further details on the determination of the energy barriers are provided in the Supplementary Materials.

## Supplementary Materials

### This PDF file includes:

Supplementary Text Figs. S1 to S18 Tables S1 to S4 References
