# Supplementary material for: Approaching infinite selectivity in membrane-based aqueous lithium extraction via solid-state ion transport
Source: Sci Adv. 2025 Feb 28;11(9):eadq9823. doi: 10.1126/sciadv.adq9823 (PMC11870030; doi:10.1126/sciadv.adq9823)
Supplement: Supplementary file 1 — Supplementary Text Figs. S1 to S18 Tables S1 to S4 References [file sciadv.adq9823_sm.pdf]

Science Advances
AAAS

Supplementary Materials for

Approaching infinite selectivity in membrane-based aqueous lithium extraction via solid-state ion transport

Sohum K. Patel *et al.*

Corresponding author: Menachem Elimelech, menachem.elimelech@rice.edu

Sci. Adv. **11**, eadq9823 (2025)
DOI: 10.1126/sciadv.adq9823

This PDF file includes:

Supplementary Text
Figs. S1 to S18
Tables S1 to S4
References

Electrodialysis Cell Design

A custom three-membrane/four-compartment electrodialysis cell was designed and fabricated, as shown in fig. S1A. Specifically, anion exchange membranes were utilized to separate the electrode rinse compartments from the inner serpentine flow channels. The inner flow channels serve as the feed and receiving channels, which make contact with the central membrane of interest (i.e., the SSE or CEM).

Acrylic plates (3 inch × 3 inch × 0.5 inch) were used as the endplates. Platinum coated (2.5 microns) titanium mesh electrodes (MSE Supplies) were used as both the anode and cathode and were inserted into appropriately sized grooves (1 mm depth) in the acrylic endplates. An Ultra-Corrosion-Resistant Grade 2 titanium rod (McMaster Carr) was inserted through the acrylic endplates via a compression fitting and pushed against the electrodes, ensuring an electrically conductive connection between the potentiostat leads (clipped to the titanium rod) and the electrodes. The electrode rinse flow channels were cut as open (1 inch × 1 inch) squares into 0.5" thick Garolite, while the inner flow channels (which run along the central membrane) were cut as serpentine paths into 0.05 inch thickness Garolite. The inner flow channels were kept as thin as possible to minimize solution resistance. For the measurement of membrane resistance and energy barriers, the serpentine inner flow paths were replaced with 1.25 inch depth square open channels, which were designed to allow for the insertion of Luggin capillaries (2 mm inner diameter).

Each of the membranes was sandwiched between a pair of EDPM gaskets to ensure sealing and to evenly distribute the force upon cell compression (particularly important for the fragile SSE material). Holes were drilled into the gaskets and flow channels to direct the fluid flow and create four hydraulically separate flow paths (i.e., two electrode rinse solution flow paths, feed solution flow path, and receiving solution flow path). The flow channels and holes were precisely cut into each material using a CNC milling machine. Upon assembly, the cell was compressed using stainless steel socket head screws.

Concentration Gradient Driven Transport Experiments

To assess the potential for purely diffusive transport of lithium through the SSE, the ED flow cell was utilized without the application of an external potential. A large transmembrane concentration gradient of lithium chloride was maintained across the SSE by recirculating 20 mL solutions of 0.5 mol L^{-1} LiCl and ultrapure deionized water (> 18.2 megaohm cm) through the feed and receiving solutions, respectively. A 150 mL solution of 0.5 mol L^{-1} $MgSO_4$ was recirculated through the electrode rinse compartments to simultaneously assess concentration gradient driven transport through the AEM into the receiving compartment. The flowrate of the feed and receiving solutions was fixed at 1 mL min^{-1}, while the flowrate of the electrode rinse solution was 8 mL min^{-1}. Samples (0.2 mL) were periodically collected from the receiving solution and tested for cation composition using ion chromatography.

Water Transport and Stability Testing

The interaction of water with the SSE material was assessed through water uptake experiments. Four SSE fragments of varying mass were weighed prior to being immersed in 50 mL of deionized water. After the SSE fragments had been soaked for 48 hours, they were removed from the water, gently patted dry with a Kimwipe, and immediately weighed.

The stability of the SSE material in water was also evaluated by conducting lithium ion leaching experiments. Three fragments of the SSE were placed in 10.0 mL of ultrapure deionized water (> 18.2 megaohm cm). Aliquots (0.2 mL) of the water were periodically taken over the course of one week and lithium concentration was measured using ion chromatography.

A custom-built diffusion cell was utilized to evaluate the relative water permeability of the SSE and CEM (fig. S5). Specifically, a membrane coupon was clamped between two 60 mL glass compartments. One compartment was filled with an aqueous solution of 0.5 mol L^{-1} sucrose, while the other solution was filled with ultrapure deionized water (> 18.2 megaohm cm). The volume of water transferred from the deionized water solution to the sucrose solution was determined by reading the level on a syringe attached to the sucrose solution compartment. The exposed membrane area available for water transport was 1.77 cm^2.

Determination of Energy Barriers

Transition state theory was applied to determine the energy barriers for lithium ion transport in the SSE and CEM. As described in the Materials and Methods section of the manuscript, the permeability defined by Equation 6 can be substituted into Equation 5, resulting in the following relation, where σ_m is the membrane conductivity, h is Planck's constant, R is the ideal gas constant, λ is the molecular jump length, k_B is the Boltzmann constant, T is the absolute temperature, ΔS is the entropy of activation, ΔH is the enthalpic barrier for permeability, and the subscript i refers to either the SSE or CEM.

$$\frac{\sigma_m hR}{{\lambda_i}^2 k_B F^2 C_i} = e^{\Delta S/R} e^{-\Delta H/RT} \qquad \text{(S1)}$$

The linearized form of the equation is given by

$$ln\left(\frac{\sigma_m hR}{{\lambda_i}^2 k_B F^2 C_i}\right) = ln(\sigma_m \alpha_i) = -\frac{1}{T}\left(\frac{\Delta H}{R}\right) + \frac{\Delta S}{R} \qquad \text{(S2)}$$

where α is a parameter that lumps all constants. By plotting $ln(\sigma_m \alpha)$ versus $\frac{1}{T}$, ΔH can be extracted from the slope. To determine the entropic energy barrier (i.e., in units of energy), it is necessary to assume that the entropy of activation is constant over the studied temperature range. Accordingly, the entropic energy barrier can be approximated as $-\bar{T}\Delta S$ where $\bar{T}$ is the average temperature for the studied range. The Arrhenius plots for the SSE and CEM are shown in Figure S8 and the values for each of the parameters and energy barriers are provided in Table S1 and Table S2.

The average molecular jump length in the SSE can be reasonably approximated as 4.0 Å through evaluation of the crystal structure, along with the assumption that lithium ions primarily migrate via hops between lattice sites and metastable interstitial sites (*31, 71*). In contrast, the elementary ion jumps in the CEM are more challenging to predict due to the relatively stochastic nature of ion transport through ion-exchange membranes. Previous work which aimed to model the activation behavior of ion-exchange membranes, nonetheless, has suggested that the ion jumps in ion exchange membranes may effectively be treated as those in bulk water, whereby each hop is effectively separated by the diameter of one water molecule (*72*). Notably, the use of this assumption has also recently been experimentally validated, showing good agreement over a wide range of tested membranes and solutes (*49*). Thus, we also assumed the CEM molecular jump length to be 2.8 Å.

The concentration of lithium ions inside the CEM (C) is approximated using standard ion-exchange capacity measurement techniques described in the literature (*49, 73*). Specifically, a fresh CEM coupon (in Na-form) was brought into Li-form by soaking in 500 mM LiCl solution (50 mL) for > 24 hours. The solution was replaced three times to ensure complete ion-exchange.

After converting the CEM to Li-form, the CEM was converted back to Na-form following the same procedure, using 0.1 M NaCl solutions. The total quantity of lithium ions eluted was determined by summing the amount eluted into each of the NaCl elution solutions (measured with ICP-MS). The concentration in the membrane is calculated by normalizing the total moles of lithium ions eluted by the hydrated membrane geometric volume (determined by measuring the hydrated membrane thickness with a digital micrometer).

The concentration of lithium inside the SSE was approximated according to the stoichiometry reported by the manufacturer (Li_2O-Al_2O_3-SiO_2-P_2O_5-TiO_2-GeO_2). Specifically, the lithium ion concentration is determined according to the molecular weight of the SSE structure (MW_{SSE}), and the mass (m_{SSE}), area (A_m), and thickness (δ_m) of a fresh SSE membrane coupon.

$$C_{SSE} = \frac{m_{SSE}}{MW_{SSE}} \times \frac{2\ mol\ Li}{1\ mol\ SSE} \times \frac{1}{A_m \delta_m} \quad \text{(S3)}$$

The experimentally derived lithium ion concentration in the SSE provided by (Equation S3) was also compared to a purely theoretical calculation based on the molecular structure and lattice volume. Particularly, we assumed that the doped material utilized in this study has the same rhombohedral lattice unit cell dimensions as $LiTi_2(PO_4)_3$. Specifically, the $LiTi_2(PO_4)_3$ lattice has (a,b,c) dimensions of (8.623 Å, 8.623 Å, 21.081 Å) with angles between the bc, ac, and ab edges of 90.0°, 90.0°, and 120.0°, respectively. The unit cell volume (V_{uc}) for a rhombohedral lattice is given by

$$V_{uc} = \frac{\sqrt{3}}{2} a^2 c \quad \text{(S4)}$$

where a and c are the respective unit cell dimensions. Hence, the theoretical lithium ion concentration within the SSE can be calculated by normalizing the number of lithium ions (N_{Li}) in a Li_2O-Al_2O_3-SiO_2-P_2O_5-TiO_2-GeO_2 unit cell (i.e., 12 lithium ions per unit cell) by the unit cell volume.

$$C_{SSE} = \frac{N_{Li}}{V_{uc}} \quad \text{(S5)}$$

Using the experimental approach described by Equation S3, we obtain a C_{SSE} of 13.15 mol L^{-1}, while the theoretical approach of Equation S5 gives a C_{SSE} of 14.7 mol L^{-1}. Notably, the experimental and theoretical values show good agreement, with the discrepancy likely arising from the practical SSE membranes containing finite grain boundary volume, which is not reflected in the theoretical calculation. Hence, for the determination of energy barriers, we use the experimentally derived value.

Characterization of SSE

Characterization was performed on a pristine SSE and an SSE which had been utilized in a long-term (fifty hour) electrodialysis experiment with 10 mM LiCl and 10 mM NaCl in the feed solution. Before characterization, the SSE which had been used in the competitive ion transport experiment was thoroughly washed with ultrapure deionized water and gently patted dry using a Kimwipe.

A Hitachi SU7000 field emission scanning electron microscope (FE-SEM) with an attached Princeton Gamma Tech EDS detector was utilized to map the elemental composition of the SSE

cross-section. A 10 kV electron beam was used with variable pressure operation mode at 10 Pa to avoid applying a conductive coating to the SSE. A Rigaku SmartLab X-ray diffractometer (XRD) was utilized to assess the crystalline structure of the SSE samples. Diffraction patterns were collected from 10° to 90° with a step size of 0.01° using Cu K-α radiation.

Molecular Dynamics Simulations
In order to simulate the process of ions passing between two aqueous solutions through the SSE, a composite structure model was constructed using Materials Studio 2020. Three dimensional periodic models of LiCl solutions were constructed using the Amorphous Cell module, with dimensions of 44.8 Å × 44.1 Å × 38.2 Å and an initial density of 1 g/cm^3. Three simulation boxes (each containing water molecules) were assessed: pure LiCl, LiCl + NaCl, and LiCl + $MgCl_2$. The initial concentrations of sodium and magnesium ions in solution were equal to that of lithium in the relevant simulations. The thickness of the SSE is set to 8.6 Å, and a layer-by-layer structure is established through the build function. The SSE membrane is based on the included .cif file for the $LiTi_2(PO_4)_3$ lattice structure.

The molecular structure of the simulation box was optimized using the Forcite Module with COMPASSII as the force field. During the geometric optimization, the convergence threshold for maximum energy change, maximum force, and maximum displacement were set to 0.001 kcal/mol, 0.5 kcal/mol/Å, and 0.015 Å, respectively. To release the internal stress in the system, a molecular dynamics (MD) simulation was performed under the NPT ensemble for 100 ps at 0.0001 GPa and 298 K until the density was stable over time. Further optimization of the system involved an MD simulation under the NVT ensemble for 100 ps. The simulation of ion-solvent interactions and ion passage through the SSE involved the calculation of electrostatic interaction using Ewald and van der Waals force using atom base. Precise Nose-Hoover temperature control mode and Berendsen pressure control mode were used in the simulation, and the concentration of ions permeating the membranes was calculated at the end of the MD simulation. All the simulations were conducted at the temperature of 298 K with a step size of 0.5 fs.

PoreBlazer Simulations
To investigate the size exclusion based mechanism of the SSE, PoreBlazer v4.0 (*37*) was utilized on the $LiTi_2(PO_4)_3$ lattice. The Universal Force Field (UFF) was applied throughout all the simulations. The simulation cell was specified to have (a,b,c) dimensions of (8.623 Å, 8.623 Å, 21.081 Å). The angles between the bc, ac, and ab edges of the unit cell were provided as 90.0°, 90.0°, and 120.0°, respectively. The size of the spherical probe was varied from 1.0 Å to 2.0 Å, and the fraction of the free volume occupiable by the probe (f_{probe}) was determined as

$$f_{probe} = \frac{V_{oc}}{V_{geom}} \tag{S6}$$

where V_{oc} is the occupiable volume by the probe and V_{geom} is the total geometric volume of the interstitial space in the lattice (calculated as 286.2 Å^3). The probe accessible sites were visualized by overlaying the probe occupiable volume output .xyz files with the $LiTi_2(PO_4)_3$ unit cell .xyz file in VESTA (*70*).

Approximation of Li/Mg Selectivity

Throughout all the competitive ion transport experiments, no sodium or magnesium flux was detected by ICP-MS. However, in order to quantify a lithium-magnesium selectivity to compare our results with reported values in the literature, we used the limit of detection of the ICP-MS to estimate a selectivity factor. As shown in fig. S10, a calibration curve was generated using various concentrations of magnesium ranging from 0.1 ppb to 100 ppb. To closely match the composition of the samples from the competitive ion transport experiments and account for potential interference in measurement from co-occurring ions, all the standards were prepared in a background of 10 ppm potassium chloride and 0.5 ppm lithium chloride. The calibration curve showed excellent linear fit across all the prepared standards, including the lowest standard of 0.1 ppb. Hence, we assumed the limit of detection of magnesium to be ~0.1 ppb, though it may be even lower on the Nexion 5000 ICP-MS utilized. The reported lithium-magnesium selectivity ($S_{Li/Mg}$) in Fig. 5A corresponds to the multi-salt experiments conducted with 10 mM Li and 10 mM Mg and was determined as

$$S_{Li/Mg} = \frac{\Delta C_{Li} C_{f,Mg}}{\Delta C_{Mg} C_{f,Li}} = \frac{\Delta C_{Li}}{LOD_{Mg} \times DF} \tag{S7}$$

where ΔC is the change in the concentration of the indicated species (i.e., the subscript) in the receiving solution over the duration of the experiment, C_f is the concentration of each species in the feed solution, LOD_{Mg} is the detection limit of magnesium, and DF is the dilution factor applied when preparing the samples from the multi-salt experiments for ICP-MS (i.e., 50).

Calculation of Specific Energy Consumption

The specific energy consumption (SEC) for lithium extraction is defined as the energy consumed per mole of lithium extracted. For the SSE electrodialysis process, the SEC is given by

$$SEC = \frac{E \int_0^t I \, dt}{A_m \int_0^t J_{Li} \, dt} = \frac{E \int_0^t I \, dt}{V \Delta C_{Li}} \tag{S8}$$

where E is the applied potential, I is the current, t is the duration of the experiment, J_{Li} is the lithium ion flux, V is the volume of the receiving solution, and ΔC_{Li} is the change in the lithium ion concentration in the receiving solution over the duration of the experiment.

For the pressure-driven nanofiltration membranes referenced from the literature, the SEC is determined as

$$SEC = \frac{Q \Delta P}{A_m J_{Li}} \tag{S9}$$

where Q is the flowrate of pressurized feedwater to the membrane module and ΔP is the applied hydraulic pressure. The lithium flux, membrane area, flowrate, and hydraulic pressure reported in each study were utilized.

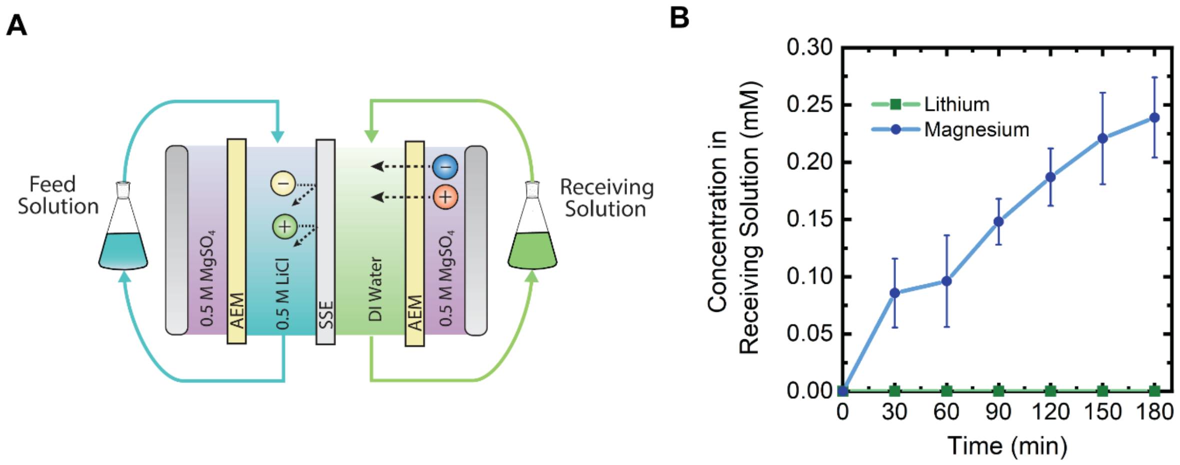

Fig. S1. Diffusion experiments with the SSE. (A) Schematic showing the experimental configuration. The electrodialysis cell was utilized but no electric potential was applied. The feed and receiving solutions were 0.5 mol L^{-1} LiCl and deionized water, respectively. A 0.5 mol L^{-1} $MgSO_4$ solution was circulated through the electrode rinse compartments. All solutions were pumped at a flowrate of 1 mL min^{-1}. (B) The lithium (green) and magnesium (blue) concentrations in the receiving solution over three hours. Lithium ions did not pass through the SSE, while magnesium ions diffused through the AEM.

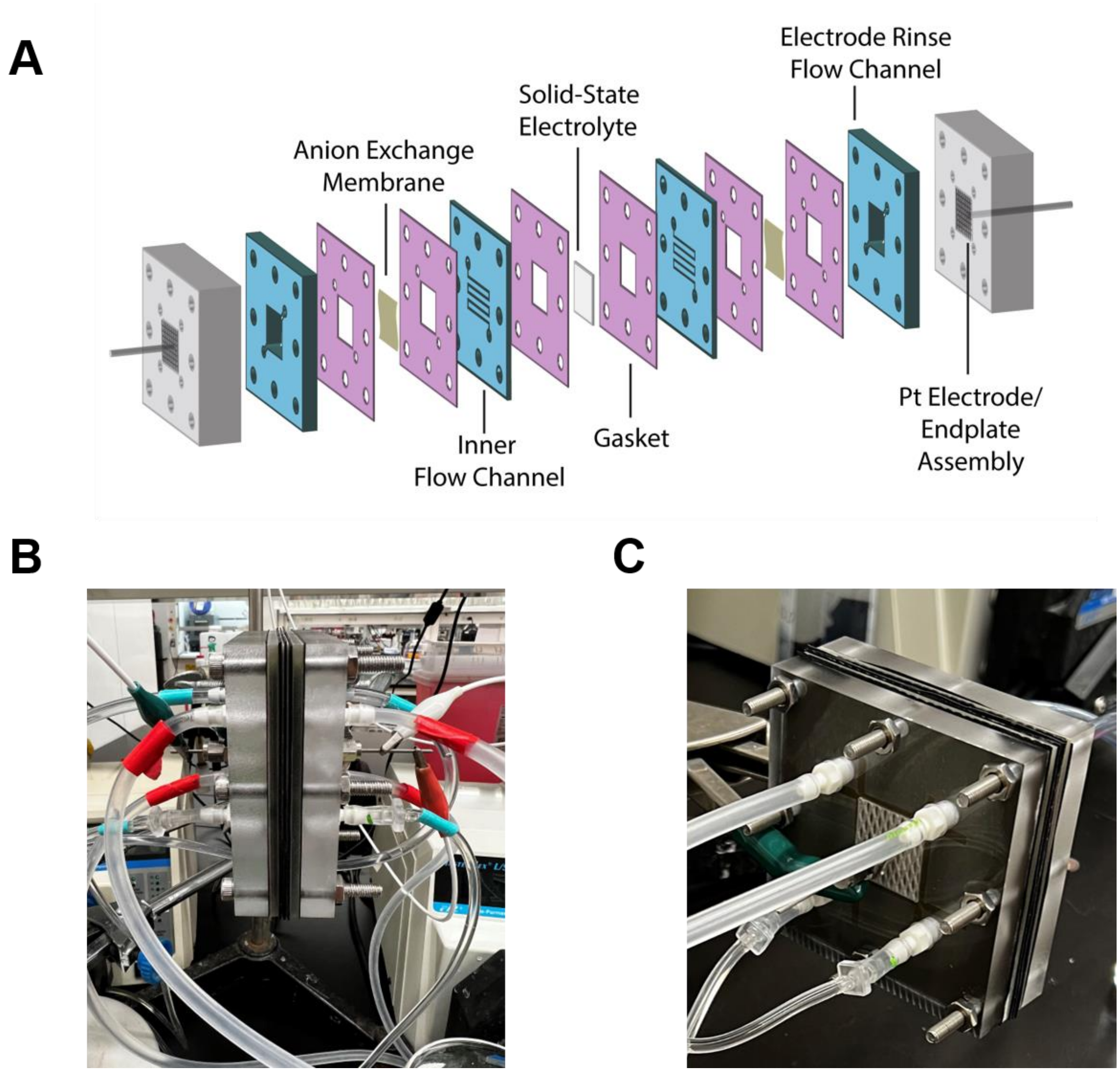

Fig. S2. Design of the custom-built electrodialysis flow-cell utilized to assess the SSE and CEM performance. (A) Illustration of the design and components of the custom-built electrodialysis flow cell utilized to assess SSE performance. The feed and receiving solutions flow across the SSE via the serpentine inner flow channels, while the external flow channels serve as electrode rinse compartments. (B) Side profile of the cell showing the stacked components in Fig. 1A. The stack consists of serpentine flow paths that pass along the central membrane and open square flow channels for the electrode rinse solutions. EPDM gaskets are used to seal the individual components together. The potentiostat leads are connected to the titanium rods which extend from the platinum coated titanium electrodes. (C) Alternate angle showing the face of the cell, through which the platinum coated titanium mesh electrode can be viewed.

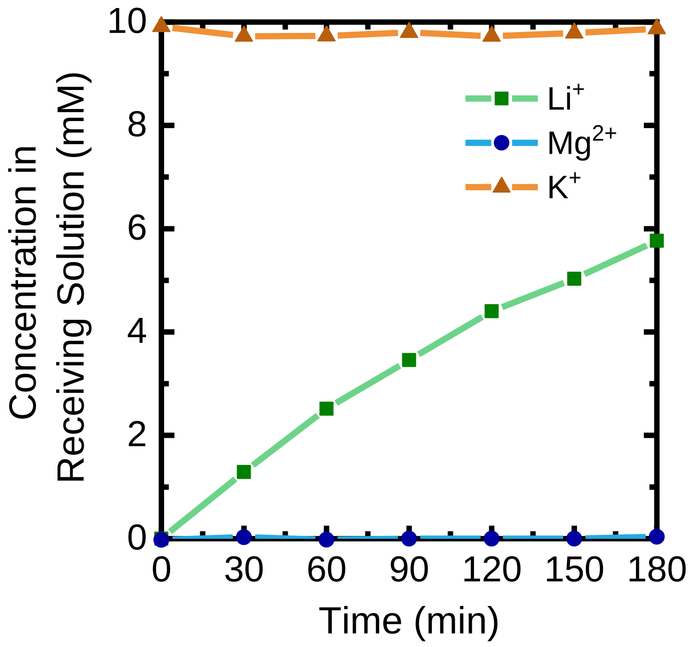

Fig. S3. Concentration in receiving solution over an electrodialysis experiment using the SSE. The lithium (green) magnesium (blue), and potassium (orange) concentrations in the receiving solution over a three hour electrodialysis experiment conducted at a constant potential of 4V. The feed solution was 10 mM LiCl, the receiving solution was 10 mM KCl, and the electrode rinse solution was 10 mM $MgSO_4$.

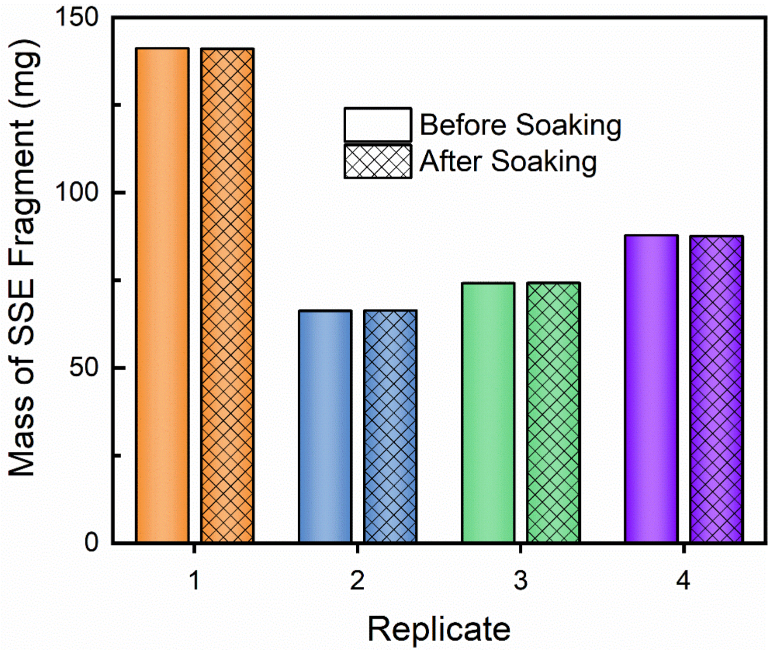

Fig. S4. Water uptake experiments performed using fragments of the SSE. Four replicates were performed with various SSE fragments, and the mass of the fragment was measured before and after soaking in deionized water for two days.

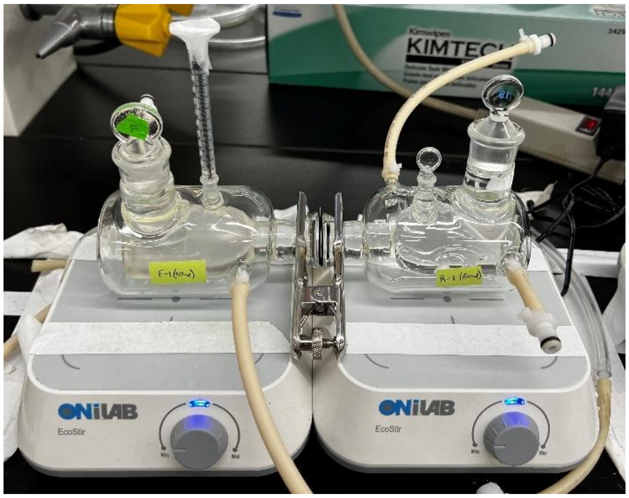

Fig. S5. Experimental setup for evaluating transmembrane water transport. A membrane coupon (either SSE or CEM) is clamped between two 60 mL solution compartments. One compartment was filled with an aqueous solution of 0.5 mol L^{-1} sucrose, while the other solution was filled with deionized water. The level on the syringe attached to the sucrose solution compartment was monitored to determine the transfer of water across the membrane.

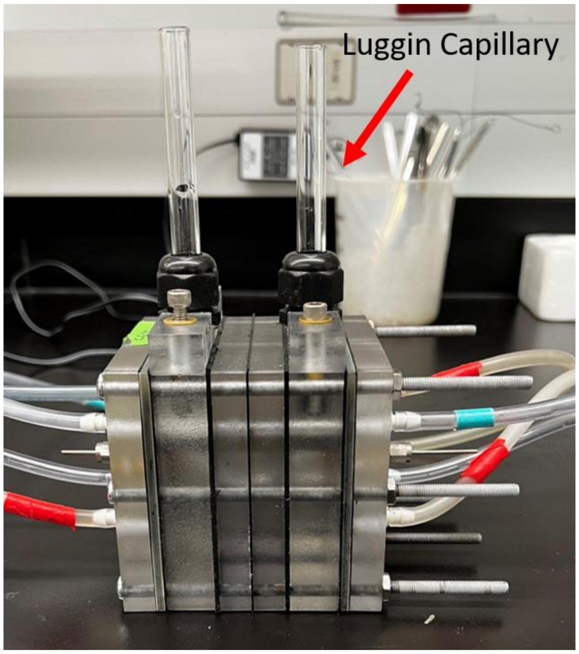

Fig. S6. Custom-built electrodialysis flow-cell utilized to measure the conductivity and energy barriers of the SSE and CEM. The inner flow channels in the more compact ED flow cell were replaced with thicker flow channels carved into acrylic plates. Luggin capillaries were inserted into to the acrylic plates through compression fittings, and the capillary tips were oriented to be close to the central membrane surface, without making direct contact. The Luggin capillaries were filled with 0.5 M KCl and Ag/AgCl reference electrodes were placed in each capillary to measure the potential difference across the central membrane. Varying temperature studies were performed by submerging all solutions and the cell (up the height of the titanium rods) in a water bath (with continuous recirculation of the heated water).

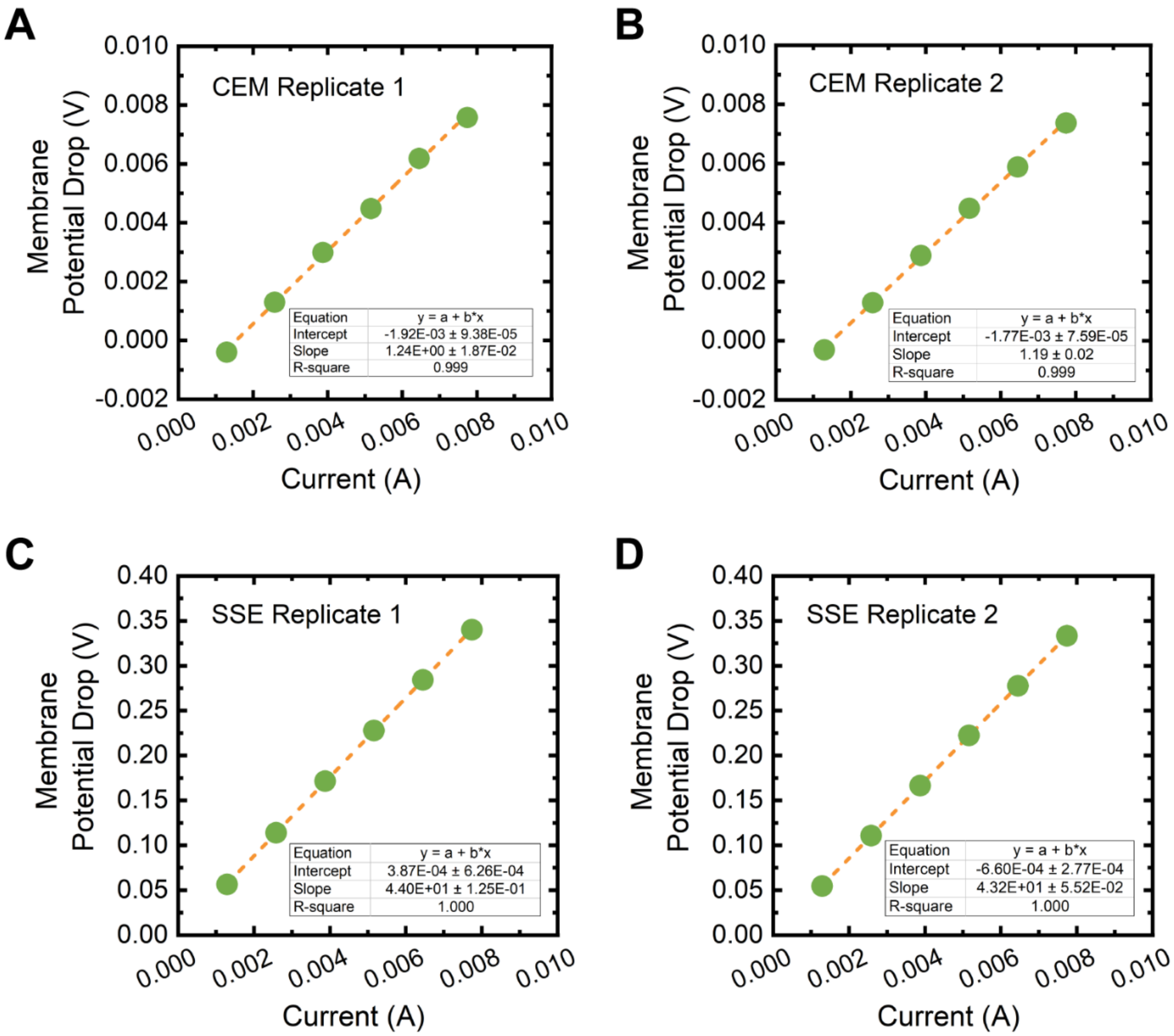

Fig. S7. Measured membrane potential drop as a function of the applied current for determination of membrane resistance. The CEM replicates are shown by (A) and (B), while the SSE replicates are shown by (C) and (D). The green points represent the potential drop measured across the membrane, and the orange dashed line shows the linear fit of each data set. The fitting parameters (i.e., slope and intercept) are shown in the table within each plot. The slope of the line is equal to the membrane resistance. The experiments were conducted in the custom-built electrodialysis cells with Luggin capillaries.

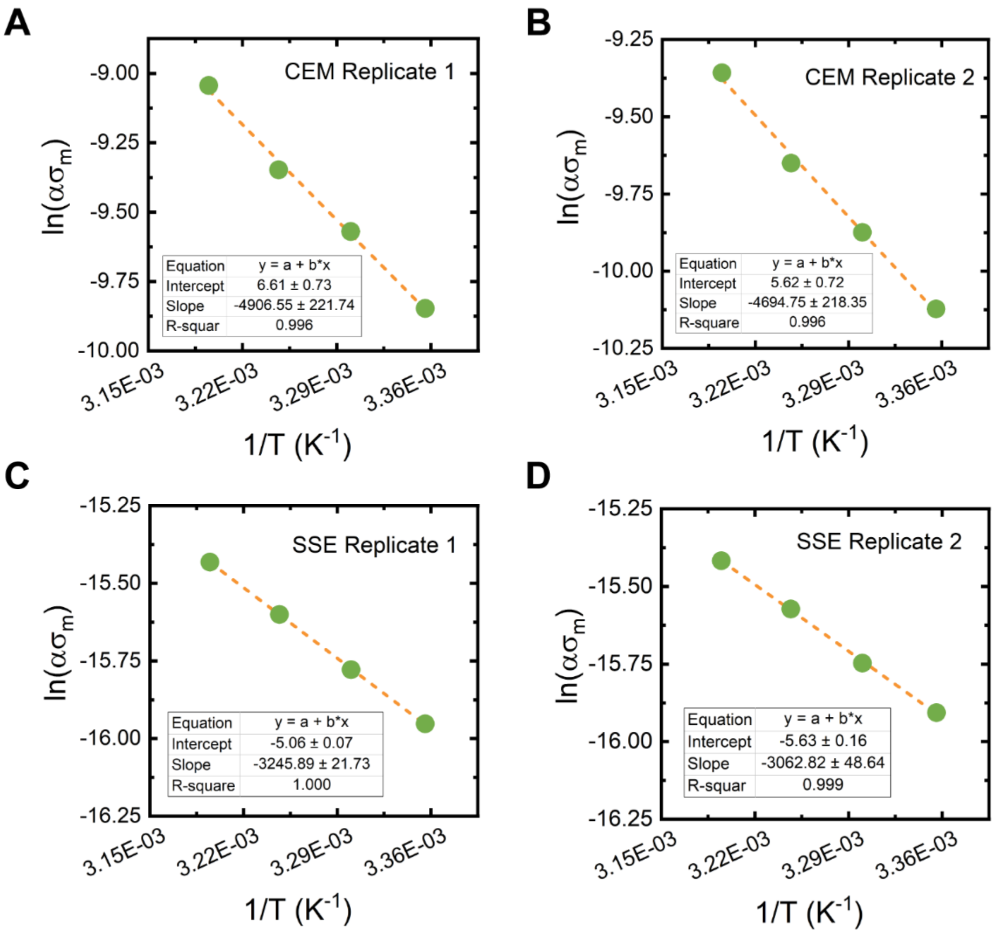

Fig. S8. Arrhenius plots for determination of energy barriers. The CEM replicates are shown by (A) and (B), while the SSE replicates are shown by (C) and (D). The green points show the natural logarithm of the measured membrane conductivity $(\boldsymbol{\sigma_m})$ multiplied by the membrane specific lumped parameter (α). The membrane conductivity was measured at various temperatures ranging from 25 ^{o}C to 40 ^{o}C. The orange dashed line shows the linear fit of each data set. The fitting parameters (i.e., slope and intercept) are shown in the table within each plot.

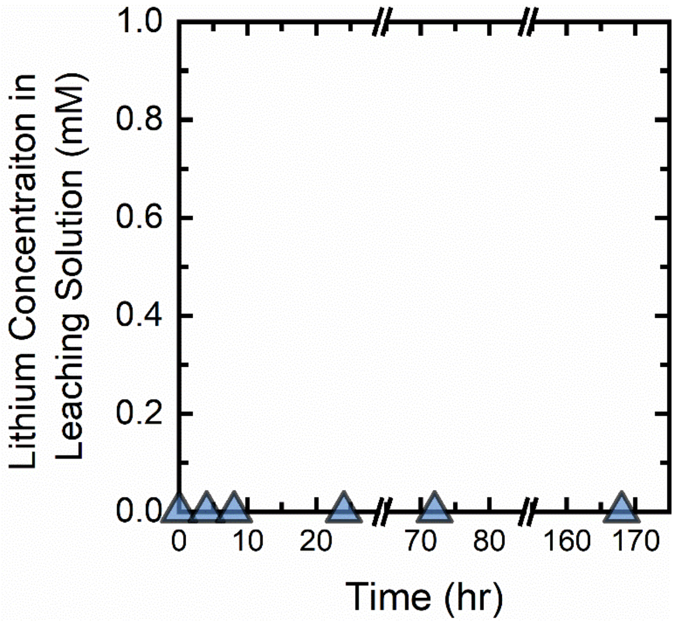

Fig. S9. The measured concentration of lithium in the leaching experiments performed with the SSE. The SSE was immersed in deionized water for one week. Ion chromatography was unable to detect any lithium ions in solution over the entire period.

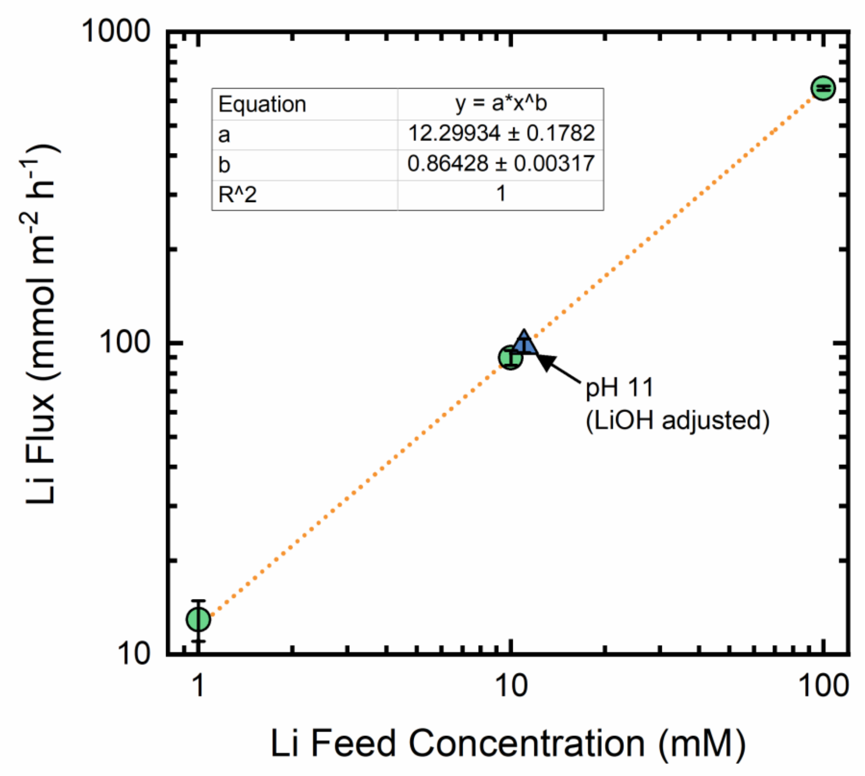

Fig. S10. Effect of pH adjustment with lithium hydroxide on lithium flux. The lithium flux across the SSE as a function of the feed lithium concentration is shown. The green points are taken from the SSE data presented in Fig. 1E of the manuscript. A power law fitting is applied to these points as shown by the dashed orange line and equation in the plot. The experimentally obtained lithium flux for the experiments conducted at pH 11 (pH was adjusted with LiOH) is shown by the blue triangle, showing good fit with the expected flux increase.

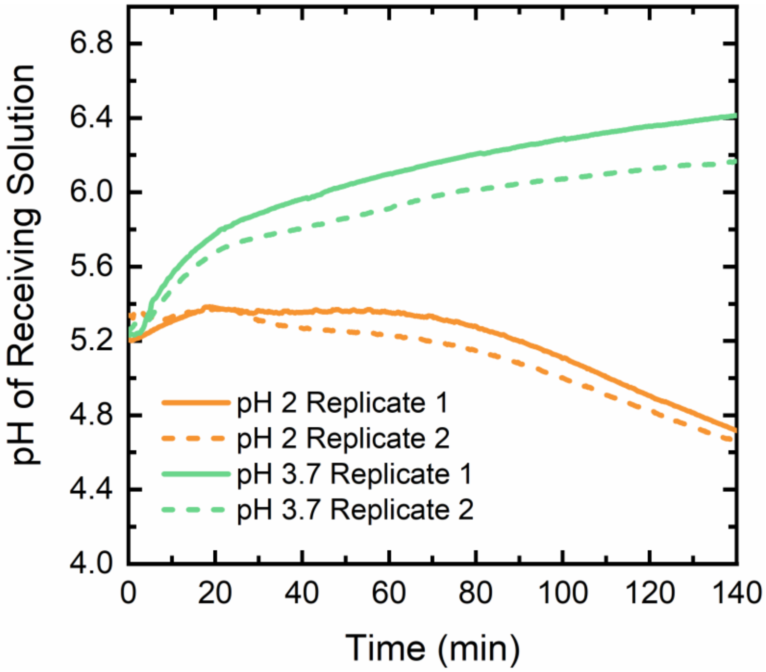

Fig. S11. The pH of the receiving solution over the duration of the SSE electrodialysis experiments conducted at pH 2 (orange lines) and pH 3.7 (green lines). Each line shows a different replicate. The feed solution was 10 mM LiCl, the receiving solution was 10 mM KCl, and the electrode rinse solution was 10 mM $MgSO_4$. The pH of the feed solution was adjusted by dosing HCl.

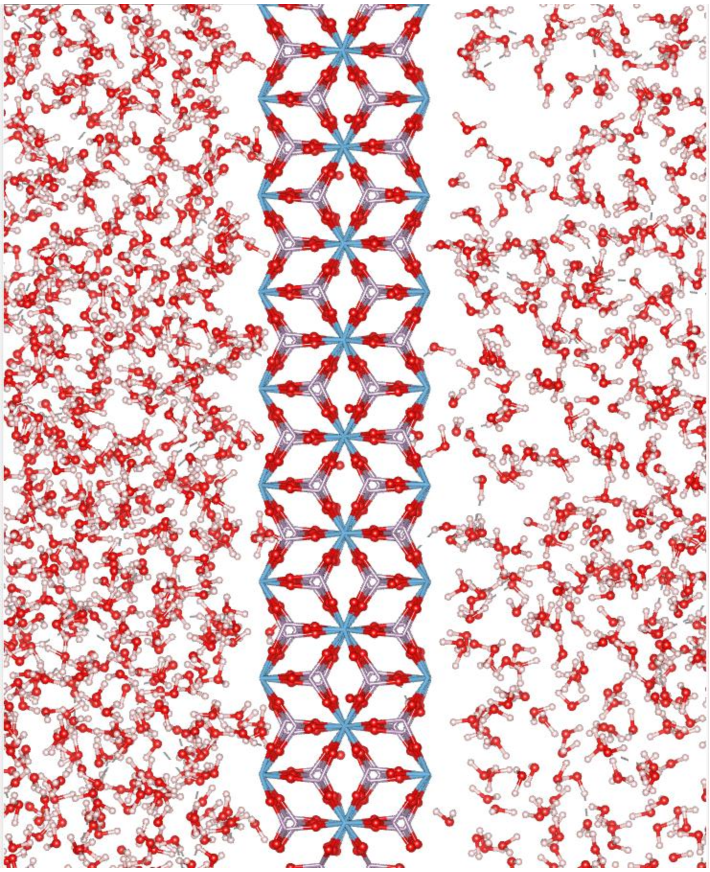

Fig. S12. Water molecule exclusion by the SSE from molecular dynamics simulations. Snapshot at the end of the molecular dynamics simulation showing only water molecules and the SSE structure. No water molecules exist within the SSE structure.

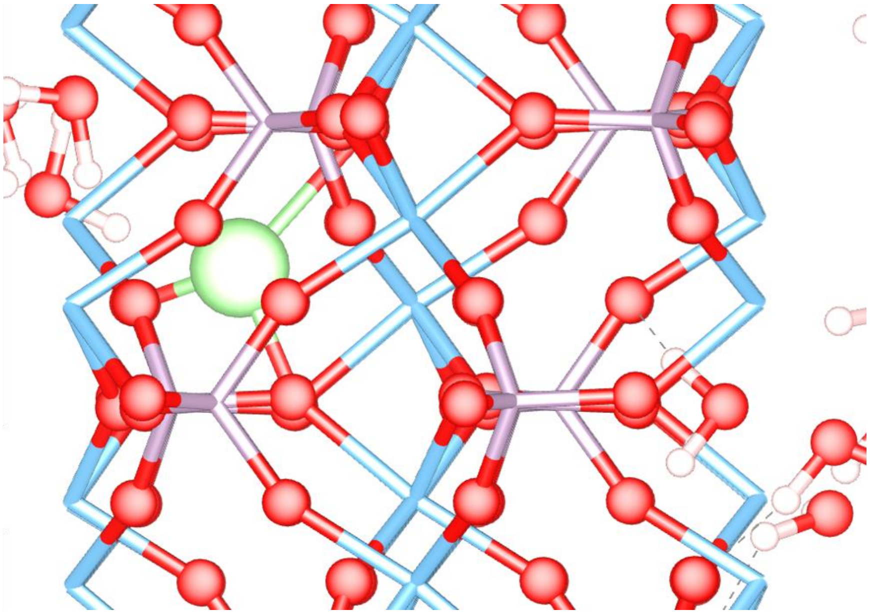

Fig. S13. Anhydrous transport of lithium ion through the SSE structure from molecular dynamics simulation. Snapshot at the end of the molecular dynamics simulation showing a single lithium ion (green sphere) transporting through the SSE structure. For clarity, other lithium ions are not shown. As the lithium ion traverses the SSE, it forms coordination bonds with oxygen atoms within the SSE.

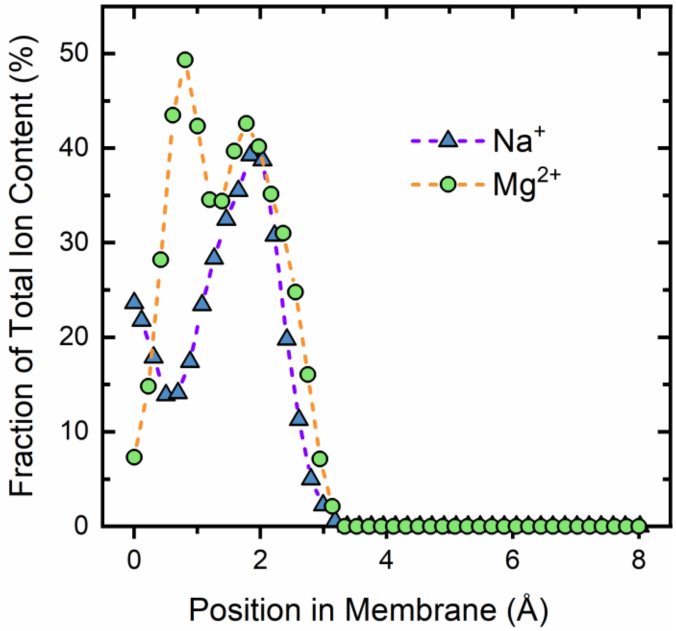

Fig. S14. Molecular dynamics simulation results showing the fraction of the total ion content made up of the competing ion (i.e., sodium or magnesium) at various positions along the length of the simulated SSE membrane. The blue triangles and purple line indicate the results from the simulated system containing lithium and sodium in the feed solution, while the green circles and orange lines show the data from the simulation where lithium and magnesium ions were in the feed solution. Only the sodium and magnesium ion fraction are shown while the remainder of the content is made up of lithium ions.

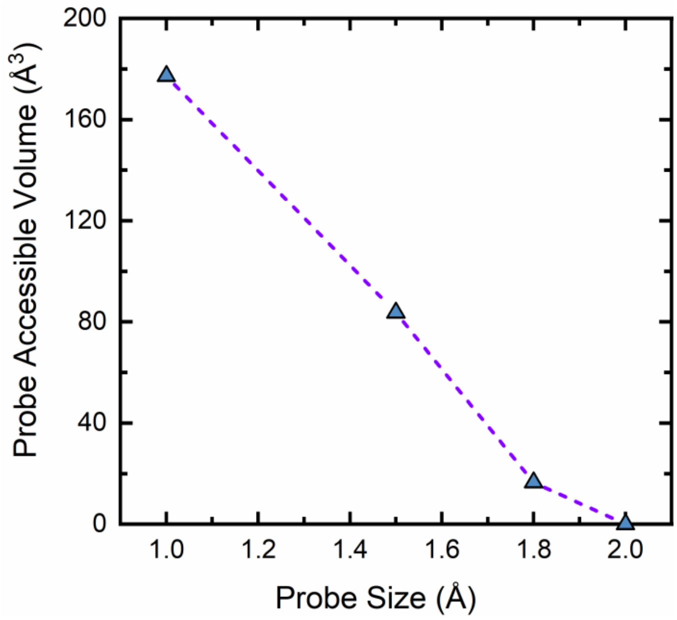

Fig. S15. PoreBlazer simulation results showing the accessible volume of a $LiTi_2(PO_4)_3$ unit cell for various sized probes. The results shown correspond to the visual representation in Fig. 4B.

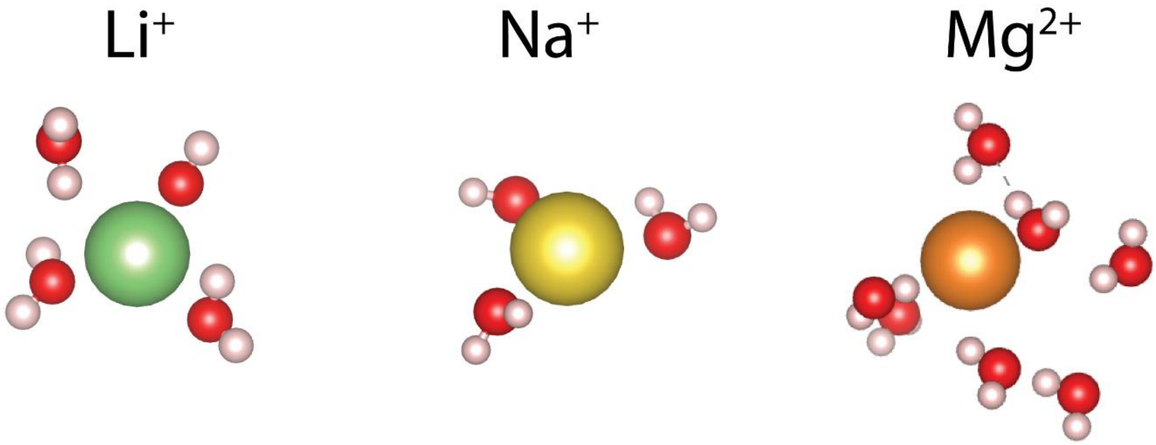

Fig. S16. Hydration state of ions in bulk solution from molecular dynamics simulation. The water molecules which exist within the expected hydrated diameter of each ion (according to table S3) are shown.

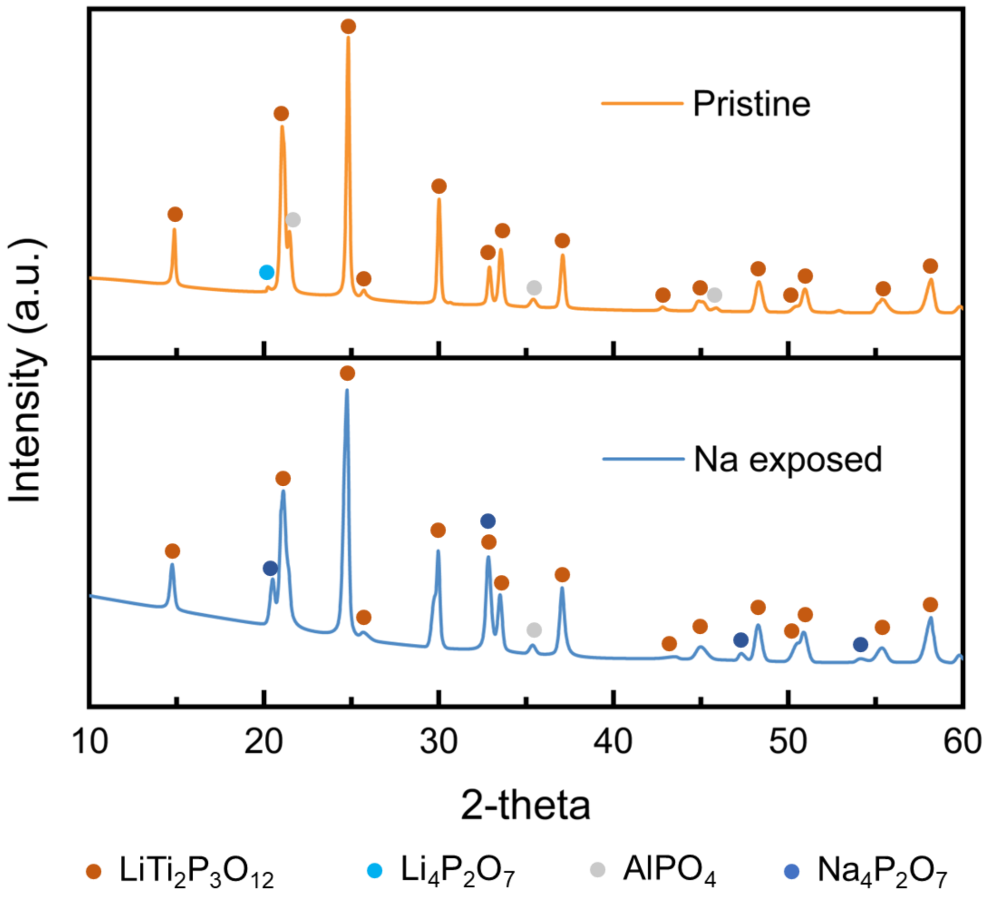

Fig. S17. X-ray diffraction patterns of the SSE. The orange line shows the diffraction pattern for the pristine SSE (i.e., before being used in any experiments), while the blue line indicates the diffraction pattern for the SSE after it had been utilized in a long-term multi-salt electrodialysis experiment in which the feed solution consisted of 10 mM LiCl and 10 mM NaCl. Each of the peaks are assigned to a specific phase according to the legend provided. $Na_4P_2O_7$: JCPDS No. 10–0187. $Li_4P_2O_7$: JCPDS No. 01-087-0409. $LiTi_2P_3O_{12}$: JCPDS No. 35-0754. $AlPO_4$: JCPDS No. 04-015-7509.

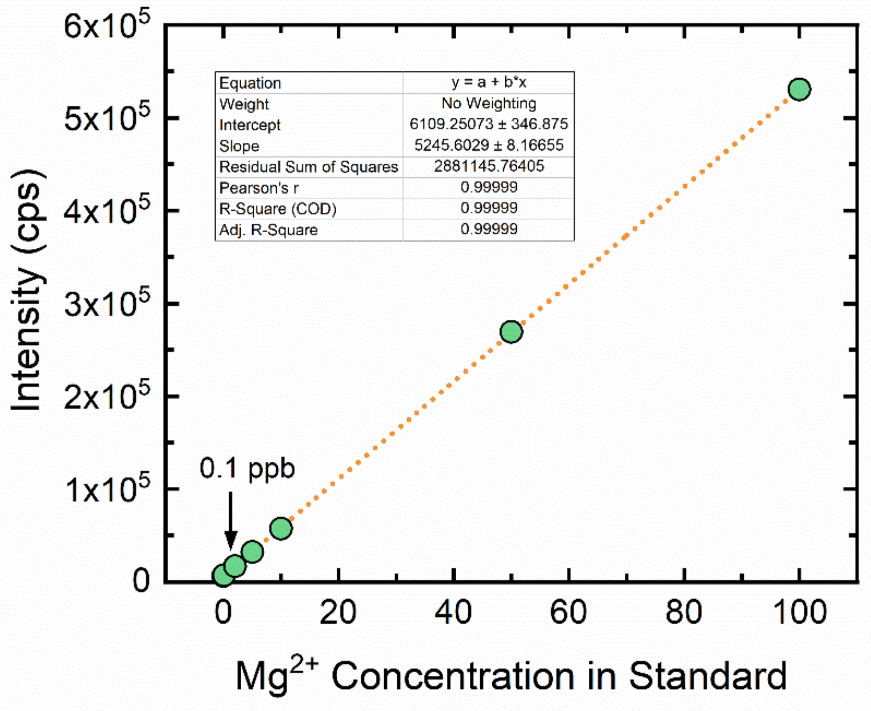

Fig. S18. Calibration curve of magnesium on the Nexion 5000 ICP-MS. The calibration standards consisted of various parts-per billion concentrations of magnesium ranging from 0.1 ppb to 100 ppb, and were prepared with a background concentration of 10 ppm potassium and 0.5 ppm lithium to closely match the matrix of tested samples from the competitive ion transport experiments. A linear fit of the data is shown, indicating excellent quantification of magnesium ions down to concentrations < 0.1 ppb. For the purpose of providing an approximate selectivity value of the SSE for comparison, 0.1 ppb was utilized as the limit of detection of the ICP-MS (and was subsequently scaled by the dilution factor of sample preparation).

Table S1. Membrane conductivity for the CEM and SSE over varying temperature for each replicate.

Membrane	Replicate	Temperature (ºC)	Conductivity (mS cm^{-1})
CEM	1	25	1.33
		30	1.75
		35	2.19
		40	2.96
	2	25	1.01
		30	1.29
		35	1.62
		40	2.17
SSE	1	25	0.058
		30	0.069
		35	0.082
		40	0.097
	2	25	0.061
		30	0.071
		35	0.085
		40	0.099

Table S2. The determined energy barriers of the CEM and SSE for each replicate along with the relevant membrane specific parameters.

Membrane	λ_i (Å)	C_i (mol L^{-1})	Replicate	ΔG (kcal mol^{-1})	ΔH (kcal mol^{-1})	$-\bar{T}\Delta S$ (kcal mol^{-1})
CEM	2.8	1.37	1	5.73	9.75	-4.01
			2	5.92	9.33	-3.41
SSE	4.0	13.1	1	9.52	6.45	3.07
			2	9.51	6.09	3.26

Table S3. The properties of the ions relevant to the competitive ion transport experiments. The crystallographic diameter (i.e., ion size in a crystalline lattice) is provided for tetrahedral (IV) and octahedral (VI) coordination environments. The hydrated diameter and hydration energy of the ions is also provided for aqueous environments.

**Ion**	**Crystallographic Diameter Coordination IV/VI (Å)(*57*)**	**Hydrated Diameter (Å)(*74*)**	**Hydration Energy (kJ mol^{-1})(*75*)**
Li^{+}	1.18/1.52	7.64	-475
Na^{+}	1.98/2.00	7.16	-365
Mg^{2+}	1.14/1.32	8.56	-1830

Table S4. The lithium flux and lithium-magnesium selectivity for various membrane materials reported in the literature, as shown in Fig. 5A.

Membrane Type	Li flux (mmol m^{-2} h^{-1})	Li/Mg Selectivity Factor
COF*(63)*	38.3	36
COF*(22)*	47.5	443
MOF*(24)*	1.08	1.63
MOF*(23)*	32.76	4
MOF*(60)*	45	25
MOF*(60)*	44.64	39
MOF*(60)*	68.4	38.5
MOF*(60)*	71.28	65
MOF*(18)*,*	3.73	1815
MOF*(62)*	12.96	3.8
Polymer*(16)*	42.37	4147
Polymer*(16)*,#	70.32	1705
Polymer*(16)*	110.88	828
Polymer*(16)*	178.49	548
Polymer*(76)*	74.02	6.1
Polymer*(76)*	58.97	3.9
Polymer*(76)*	105.30	8
Polymer*(76)*	89.66	6.7
Polymer*(76)*	67.51	4.9
Polymer*(68)*,#	204.96	78.5
Polymer*(68)*	143.66	87.2
Polymer*(68)*	399	81.1
Polymer*(59)*	428.4	1.46
Polymer*(59)*	185.04	3.83
Polymer*(59)*	160.92	5.16
Polymer*(59)*	396	1.74
Polymer*(59)*	414	0.7
SSE (this work)	**16.4**	**25704**

*The lithium flux was calculated based off the provided current density data in 0.5 M LiCl solution.
#The specific energy consumption for this data point is shown in Fig. 5B of the manuscript.

REFERENCES AND NOTES

1. D. Castelvecchi, Electric cars and batteries: How will the world produce enough? *Nature* **596**, 336–339 (2021).

2. C. Xu, Q. Dai, L. Gaines, M. Hu, A. Tukker, B. Steubing, Future material demand for automotive lithium-based batteries. *Commun. Mater.* **1**, 99 (2020).

3. M. S. Ziegler, J. E. Trancik, Re-examining rates of lithium-ion battery technology improvement and cost decline. *Energ. Environ. Sci.* **14**, 1635–1651 (2021).

4. P. Greim, A. A. Solomon, C. Breyer, Assessment of lithium criticality in the global energy transition and addressing policy gaps in transportation. *Nat. Commun.* **11**, 4570 (2020).

5. A. Kumar, H. Fukuda, T. A. Hatton, J. H. Lienhard V., Lithium recovery from oil and gas produced water: A need for a growing energy industry. *ACS Energy Lett.* **4**, 1471–1474 (2019).

6. S. E. C. Sener, V. M. Thomas, D. E. Hogan, R. M. Maier, M. Carbajales-Dale, M. D. Barton, T. Karanfil, J. C. Crittenden, G. L. Amy, Recovery of critical metals from aqueous sources. *ACS Sustainable Chem. Eng.* **9**, 11616–11634 (2021).

7. R. M. DuChanois, N. J. Cooper, B. Lee, S. K. Patel, L. Mazurowski, T. E. Graedel, M. Elimelech, Prospects of metal recovery from wastewater and brine. *Nat. Water* **1**, 37–46 (2023).

8. E. J. M. Dugamin, A. Richard, M. Cathelineau, M.-C. Boiron, F. Despinois, A. Brisset, Groundwater in sedimentary basins as potential lithium resource: A global prospective study. *Sci. Rep.* **11**, 21091 (2021).

9. P. K. Choubey, M. S. Kim, R. R. Srivastava, J. C. Lee, J. Y. Lee, Advance review on the exploitation of the prominent energy-storage element: Lithium. Part I: From mineral and brine resources. *Miner. Eng.* **89**, 119–137 (2016).

10. A. Khalil, S. Mohammed, R. Hashaikeh, N. Hilal, Lithium recovery from brine: Recent developments and challenges. *Desalination* **528**, 115611 (2022).

11. M. L. Vera, W. R. Torres, C. I. Galli, A. Chagnes, V. Flexer, Environmental impact of direct lithium extraction from brines. *Nat. Rev. Earth Environ.* **4**, 149–165 (2023).

12. C. Liu, Y. Li, D. Lin, P.-C. Hsu, B. Liu, G. Yan, T. Wu, Y. Cui, S. Chu, Lithium extraction from seawater through pulsed electrochemical intercalation. *Joule* **4**, 1459–1469 (2020).

13. Z. Y. Guo, Z. Y. Ji, J. Wang, X. F. Guo, J. S. Liang, Electrochemical lithium extraction based on “rocking-chair” electrode system with high energy-efficient: The driving mode of constant current-constant voltage. *Desalination* **533**, 115767 (2022).

14. R. Trócoli, A. Battistel, F. La Mantia, Selectivity of a lithium-recovery process based on $LiFePO_4$. *Chem.-Eur. J.* **20**, 9888–9891 (2014).

15. L. Wu, C. Zhang, S. Kim, T. A. Hatton, H. Mo, T. D. Waite, Lithium recovery using electrochemical technologies: Advances and challenges. *Water Res.* **221**, 118822 (2022).

16. Q. Peng, R. Wang, Z. Zhao, S. Lin, Y. Liu, D. Dong, Z. Wang, Y. He, Y. Zhu, J. Jin, L. Jiang, Extreme Li-Mg selectivity via precise ion size differentiation of polyamide membrane. *Nat. Commun.* **15**, 2505 (2024).

17. A. Razmjou, M. Asadnia, E. Hosseini, A. H. Korayem, V. Chen, Design principles of ion selective nanostructured membranes for the extraction of lithium ions. *Nat. Commun.* **10**, 5793 (2019).

18. Y. Guo, Y. Ying, Y. Mao, X. Peng, B. Chen, Polystyrene sulfonate threaded through a metal-organic framework membrane for fast and selective lithium-ion separation. *Angew. Chem. Int. Ed. Eng.* **55**, 15120–15124 (2016).

19. J. Lu, H. Zhang, J. Hou, X. Li, X. Hu, Y. Hu, C. D. Easton, Q. Li, C. Sun, A. W. Thornton, M. R. Hill, X. Zhang, G. Jiang, J. Z. Liu, A. J. Hill, B. D. Freeman, L. Jiang, H. Wang, Efficient metal ion sieving in rectifying subnanochannels enabled by metal-organic frameworks. *Nat. Mater.* **19**, 767–774 (2020).

20. H. Zhang, J. Hou, Y. Hu, P. Wang, R. Ou, L. Jiang, J. Z. Liu, B. D. Freeman, A. J. Hill, H. Wang, Ultrafast selective transport of alkali metal ions in metal organic frameworks with subnanometer pores. *Sci. Adv.* **4**, eaaq0066 (2018).

21. R. J. Mo, S. Chen, L. Q. Huang, X. L. Ding, S. Rafique, X. H. Xia, Z. Q. Li, Regulating ion affinity and dehydration of metal-organic framework sub-nanochannels for high-precision ion separation. *Nat. Commun.* **15**, 2145 (2024).

22. L. Hou, W. Xian, S. Bing, Y. Song, Q. Sun, L. Zhang, S. Ma, Understanding the ion transport behavior across nanofluidic membranes in response to the charge variations. *Adv. Funct. Mater.* **31**, 2009970 (2021).

23. C. Zhang, Y. Mu, W. Zhang, S. Zhao, Y. Wang, PVC-based hybrid membranes containing metal-organic frameworks for Li^+ /Mg^{2+} separation. *J. Memb. Sci.* **596**, 117724 (2020).

24. N. T. Eden, M. T. Scalzo, R. Hou, E. Ghasemiestahbanati, K. Konstas, M. R. Hill, Sulfonated metal–organic framework mixed-matrix membrane toward direct lithium extraction. *ACS Appl. Eng. Mater.* **1**, 2336–2346 (2023).

25. T. Famprikis, P. Canepa, J. A. Dawson, M. S. Islam, C. Masquelier, Fundamentals of inorganic solid-state electrolytes for batteries. *Nat. Mater.* **18**, 1278–1291 (2019).

26. J. C. Bachman, S. Muy, A. Grimaud, H. H. Chang, N. Pour, S. F. Lux, O. Paschos, F. Maglia, S. Lupart, P. Lamp, L. Giordano, Y. Shao-Horn, Inorganic solid-state electrolytes for lithium batteries: Mechanisms and properties governing ion conduction. *Chem. Rev.* **116**, 140–162 (2016).

27. B. Zhang, R. Tan, L. Yang, J. Zheng, K. Zhang, S. Mo, Z. Lin, F. Pan, Mechanisms and properties of ion-transport in inorganic solid electrolytes. *Energy Storage Mater.* **10**, 139–159 (2018).

28. S. Yang, F. Zhang, H. Ding, P. He, H. Zhou, Lithium metal extraction from seawater. *Joule* **2**, 1648–1651 (2018).

29. T. Hoshino, Innovative lithium recovery technique from seawater by using world-first dialysis with a lithium ionic superconductor. *Desalination* **359**, 59–63 (2015).

30. Z. Li, C. Li, X. Liu, L. Cao, P. Li, R. Wei, X. Li, D. Guo, K.-W. Huang, Z. Lai, Continuous electrical pumping membrane process for seawater lithium mining. *Energ. Environ. Sci.* **14**, 3152–3159 (2021).

31. B. Akkinepally, I. N. Reddy, T. J. Ko, K. Yoo, J. Shim, Dopant effect on Li^+ ion transport in NASICON-type solid electrolyte: Insights from molecular dynamics simulations and experiments. *Ceram. Int.* **48**, 12142–12151 (2022).

32. F. Bai, K. Kakimoto, X. Shang, D. Mori, S. Taminato, M. Matsumoto, Y. Takeda, O. Yamamoto, H. Izumi, H. Minami, N. Imanishi, Water-stable high lithium-ion conducting solid electrolyte of $Li_{1\ 4}Al_{0\ 4}Ge_{0\ 2}Ti_{1\ 4}.(PO_4)_3$-LiCl for aqueous lithium-air batteries. *Front. Energy Res.* **8**, 187 (2020).

33. P. Dlugolecki, P. Ogonowski, S. J. Metz, M. Saakes, K. Nijmeijer, M. Wessling, On the resistances of membrane, diffusion boundary layer and double layer in ion exchange membrane transport. *J. Memb. Sci.* **349**, 369–379 (2010).

34. J. Kamcev, R. Sujanani, E.-S. Jang, N. Yan, N. Moe, D. R. Paul, B. D. Freeman, Salt concentration dependence of ionic conductivity in ion exchange membranes. *J. Memb. Sci.* **547**, 123–133 (2018).

35. H. Fan, N. Y. Yip, Elucidating conductivity-permselectivity tradeoffs in electrodialysis and reverse electrodialysis by structure-property analysis of ion-exchange membranes. *J. Memb. Sc.* **573**, 668–681 (2019).

36. L. Sarkisov, R. Bueno-Perez, M. Sutharson, D. Fairen-Jimenez, Materials Informatics with PoreBlazer v4.0 and the CSD MOF Database. *Chem. Mater.* **32**, 9849–9867 (2020).

37. H. Mehrer, *Diffusion in Solids: Fundamentals, Methods, Materials, Diffusion-Controlled Processes* (Springer Science & Business Media, 2007), vol. 155.

38. N. Kononenko, V. Nikonenko, D. Grande, C. Larchet, L. Dammak, M. Fomenko, Y. Volfkovich, Porous structure of ion exchange membranes investigated by various techniques. *Adv. Colloid Interface Sci.* **246**, 196–216 (2017).

39. J. Kamcev, D. R. Paul, G. S. Manning, B. D. Freeman, Ion diffusion coefficients in ion exchange membranes: Significance of counterion condensation. *Macromolecules* **51**, 5519–5529 (2018).

40. H. Yasuda, C. E. Lamaze, L. D. Ikenberry, Permeability of solutes through hydrated polymer membranes. I. Diffusion of sodium chloride. *Makromol. Chem.* **118**, 19–35 (1968).

41. I. Shefer, K. Lopez, A. P. Straub, R. Epsztein, Applying transition-state theory to explore transport and selectivity in salt-rejecting membranes: A critical review. *Environ. Sci. Technol.* **56**, 7467–7483 (2022).

42. B. J. Zwolinski, H. Eyring, C. E. Reese, Diffusion and membrane permeability. *J. Phys. Colloid Chem.* **53**, 1426–1453 (1949).

43. C. L. Ritt, M. Liu, T. A. Pham, R. Epsztein, H. J. Kulik, M. Elimelech, Machine learning reveals key ion selectivity mechanisms in polymeric membranes with subnanometer pores. *Sci. Adv.* **8**), eabl5771 (2022.

44. X. Zhou, Z. Wang, R. Epsztein, C. Zhan, W. Li, J. D. Fortner, T. A. Pham, J.-H. Kim, M. Elimelech, Intrapore energy barriers govern ion transport and selectivity of desalination membranes. *Sci. Adv.* **6**, eabd9045 (2020).

45. P. E. Mason, S. Ansell, G. W. Neilson, S. B. Rempe, Neutron scattering studies of the hydration structure of Li. *J. Phys. Chem. B* **119**, 2003–2009 (2015).

46. H. H. Loeffler, B. M. Rode, The hydration structure of the lithium ion. *J. Chem. Phys.* **117**, 110–117 (2002).

47. S. Varma, S. B. Rempe, Coordination numbers of alkali metal ions in aqueous solutions. *Biophys. Chem.* **124**, 192–199 (2006).

48. B. Lang, B. Ziebarth, C. Elsässer, Lithium ion conduction in $LiTi_2(PO_4)_3$ and related compounds based on the NASICON structure: A first-principles study. *Chem. Mater.* **27**, 5040–5048 (2015).

49. J. C. Díaz, J. Park, A. Shapiro, H. Patel, L. Santiago-Pagán, D. Kitto, J. Kamcev, Understanding monovalent cation diffusion in negatively charged membranes and the role of membrane water content. *Macromolecules* **57**, 2468–2481 (2024).

50. H. Fan, Y. Huang, I. H. Billinge, S. M. Bannon, G. M. Geise, N. Y. Yip, Counterion mobility in ion-exchange membranes: Spatial effect and valency-dependent electrostatic interaction. *ACS Est. Eng.* **2**, 1274–1286 (2022).

51. R. Wang, S. Lin, Membrane design principles for ion-selective electrodialysis: An analysis for Li/Mg separation. *Environ. Sci. Technol.* **58**, 3552–3563 (2024).

52. A. Martinez-Juarez, C. Pecharroman, J. E. Iglesias, J. M. Rojo, Relationship between activation energy and bottleneck size for Li^+ ion conduction in NASICON materials of composition $LiMM'(PO_4)_3$; M, M′ = Ge, Ti, Sn, Hf. *J. Phys. Chem. B* **102**, 372–375 (1998).

53. L. Zhu, Y. Wang, J. Chen, W. Li, T. Wang, J. Wu, S. Han, Y. Xia, Y. Wu, M. Wu, F. Wang, Y. Zheng, L. Peng, J. Liu, L. Chen, W. Tang, Enhancing ionic conductivity in solid electrolyte by relocating diffusion ions to under-coordination sites. *Sci. Adv.* **8**, eabj7698 (2022).

54. Z. Zou, N. Ma, A. Wang, Y. Ran, T. Song, B. He, A. Ye, P. Mi, L. Zhang, H. Zhou, Y. Jiao, J. Liu, D. Wang, Y. Li, M. Avdeev, S. Shi, Identifying migration channels and bottlenecks in monoclinic NASICON-type solid electrolytes with hierarchical ion-transport algorithms. *Adv. Funct. Mater.* **31**, 2107747 (2021).

55. R. M. DuChanois, M. Heiranian, J. Yang, C. J. Porter, Q. Li, X. Zhang, R. Verduzco, M. Elimelech, Designing polymeric membranes with coordination chemistry for high-precision ion separations. *Sci. Adv.* **8**, eabm9436 (2022).

56. R. D. Shannon, Revised effective ionic-radii and systematic studies of interatomic distances in halides and chalcogenides. *Acta Crystallogr.* **32**, 751–767 (1976).

57. M. Zhang, H. Pan, Y. Wang, J. Yang, H. Dong, P. He, H. Zhou, Research on Li^+/Na^+ selectivity of NASICON-type solid-state ion conductors by first-principles calculations. *Energy Fuel* **37**, 10663–10672 (2023).

58. R. M. DuChanois, L. Mazurowski, H. Fan, R. Verduzco, O. Nir, M. Elimelech, Precise cation separations with composite cation-exchange membranes: Role of base layer properties. *Environ. Sci. Technol.* **57**, 6331–6341 (2023).

59. N. Ul Afsar, M. A. Shehzad, M. Irfan, K. Emmanuel, F. Sheng, T. Xu, X. Ren, L. Ge, T. Xu, Cation exchange membrane integrated with cationic and anionic layers for selective ion separation *via* electrodialysis. *Desalination* **458**, 25–33 (2019).

60. T. Xu, M. A. Shehzad, D. Yu, Q. Li, B. Wu, X. Ren, L. Ge, T. Xu, Highly cation permselective metal-organic framework membranes with leaf-like morphology. *ChemSusChem* **12**, 2593–2597 (2019).

61. H. Xiao, M. Chai, A. Hosseini, A. H. Korayem, M. Abdollahzadeh, H. Ahmadi, V. Chen, D. B. Gore, M. Asadnia, A. Razmjou, UiO-66-$(COONa)_2$ membrane with programmable ionic channels for lithium ion-selective transport. *J. Memb. Sci.* **670**, 121312 (2023).

62. M. Mohammad, M. Lisiecki, K. Liang, A. Razmjou, V. Chen, Metal-Phenolic network and metal-organic framework composite membrane for lithium ion extraction. *Appl. Mater. Today* **21**, 100884 (2020).

63. F. Sheng, B. Wu, X. Li, T. Xu, M. A. Shehzad, X. Wang, L. Ge, H. Wang, T. Xu, Efficient ion sieving in covalent organic framework membranes with sub-2-nanometer channels. *Adv. Mater.* **33**, 2104404 (2021).

64. P. K. Choubey, K. S. Chung, M. S. Kim, J. C. Lee, R. R. Srivastava, Advance review on the exploitation of the prominent energy-storage element lithium. Part II: From sea water and spent lithium ion batteries (LIBs). *Miner. Eng.* **110**, 104–121 (2017).

65. K. Ooi, A. Sonoda, Y. Makita, R. Chitrakar, Y. Tasaki-Handa, T. Nakazato, Recovery of lithium from salt-brine eluates by direct crystallization as lithium sulfate. *Hydrometallurgy* **174**, 123–130 (2017).

66. T. Tran, V. T. Luong, in *Lithium Process Chemistry*, A. Chagnes, J. Światowska, Eds. (Elsevier, 2015), pp. 81–124.

67. Y. Zhang, Y. Hu, L. Wang, W. Sun, Systematic review of lithium extraction from salt-lake brines via precipitation approaches. *Miner. Eng.* **139**, 105868 (2019).

68. R. He, C. Dong, S. Xu, C. Liu, S. Zhao, T. He, Unprecedented Mg^{2+}/Li^{+} separation using layer-by-layer based nanofiltration hollow fiber membranes. *Desalination* **525**, 115492 (2022).

69. S. K. Patel, M. Qin, W. S. Walker, M. Elimelech, Energy efficiency of electro-driven brackish water desalination: Electrodialysis significantly outperforms membrane capacitive deionization. *Environ. Sci. Technol.* **54**, 3663–3677 (2020).

70. K. Momma, F. Izumi, *VESTA 3* for three-dimensional visualization of crystal, volumetric and morphology data. *J. Appl. Cryst.* **44**, 1272–1276 (2011).

71. Z. Deng, T. P. Mishra, E. Mahayoni, Q. Ma, A. J. K. Tieu, O. Guillon, J.-N. Chotard, V. Seznec, A. K. Cheetham, C. Masquelier, G. S. Gautam, P. Canepa, Fundamental investigations on the sodium-ion transport properties of mixed polyanion solid-state battery electrolytes. *Nat. Commun.* **13**, 4470 (2022).

72. S. Mafé, J. A. Manzanares, P. Ramirez, Modeling of surface bulk ionic conductivity in fixed charge membranes. *Phys. Chem. Chem. Phys.* **5**, 376–383 (2003).

73. P. Dlugolecki, K. Nymeijer, S. Metz, M. Wessling, Current status of ion exchange membranes for power generation from salinity gradients. *J. Memb. Sci.* **319**, 214–222 (2008).

74. E. R. Nightingale Jr., Phenomenological theory of ion solvation. Effective Radii of hydrated ions. *J. Phys. Chem.* **63**, 1381–1387 (1959).

75. Y. Marcus, Thermodynamics of solvation of ions. Part 5.—Gibbs free energy of hydration at 298.15 K. *J. Chem. Soc. Faraday Trans.* **87**, 2995–2999 (1991).

76. L. Wang, D. Rehman, P.-F. Sun, A. Deshmukh, L. Zhang, Q. Han, Z. Yang, Z. Wang, H.-D. Park, J. H. Lienhard, C. Y. Tang, Novel positively charged metal-coordinated nanofiltration membrane for lithium recovery. *ACS Appl. Mater. Interfaces* **13**, 16906–16915 (2021).
